# An Evaluation of the Design of Multimedia Patient Education Materials in Musculoskeletal Health Care: Systematic Review

**DOI:** 10.2196/48154

**Published:** 2024-10-15

**Authors:** Garett Van Oirschot, Amanda Pomphrey, Caoimhe Dunne, Kate Murphy, Karina Blood, Cailbhe Doherty

**Affiliations:** 1 School of Public Health, Physiotherapy & Sport Science University College Dublin Dublin Ireland; 2 Insight SFI Research Centre for Data Analytics Dublin Ireland

**Keywords:** health education, patient education, patient education materials, multimedia, musculoskeletal diseases, musculoskeletal pain, eHealth, self-management

## Abstract

**Background:**

Educational multimedia is a cost-effective and straightforward way to administer large-scale information interventions to patient populations in musculoskeletal health care. While an abundance of health research informs the content of these interventions, less guidance exists about optimizing their design.

**Objective:**

This study aims to identify randomized controlled trials of patient populations with musculoskeletal conditions that used multimedia-based patient educational materials (PEMs) and examine how design was reported and impacted patients’ knowledge and rehabilitation outcomes. Design was evaluated using principles from the cognitive theory of multimedia learning (CTML).

**Methods:**

PubMed, CINAHL, PsycINFO, and Embase were searched from inception to September 2023 for studies examining adult patients with musculoskeletal conditions receiving multimedia PEMs compared to any other interventions. The primary outcome was knowledge retention measured via test scores. Secondary outcomes were any patient-reported measures. Retrievability was noted, and PEMs were sourced through search, purchase, and author communication.

**Results:**

A total of 160 randomized controlled trials were eligible for inclusion: 13 (8.1%) included their educational materials and 31 (19.4%) required a web search, purchase, or direct requests for educational materials. Of these 44 (27.5%) studies, none fully optimized the design of their educational materials, particularly lacking in the CTML principles of coherence, redundancy, modality, and generative activities for the learner. Of the 160 studies, the remaining 116 (72.5%) contained interventions that could not be retrieved or appraised. Learning was evaluated in 5 (3.1%) studies.

**Conclusions:**

Musculoskeletal studies should use open science principles and provide their PEMs wherever possible. The link between providing multimedia PEMs and patient learning is largely unexamined, but engagement potential may be maximized when considering design principles such as the CTML.

## Introduction

### Rationale

The worldwide prevalence and burden of musculoskeletal conditions are exceptionally high, affecting 20% of the global population and accounting for 150 million disability-adjusted life years [1]. They are the second-greatest contributor to worldwide disability [2] and threaten healthy aging by limiting physical and mental capacities and functional ability [3]. The United States demonstrated one of the highest levels of age-standardized disability-adjusted life-years in musculoskeletal disorders worldwide, at over 3000 per 100,000 [4]. A multidisciplinary, multimodal approach is appropriate when managing musculoskeletal conditions [5], and a vital component is patient education [6], that is, teaching the patient [7] about their condition and management options, including nonpharmacological treatment strategies such as exercise or activity modification. Patient education empowers patients with knowledge to participate in and adhere to treatment [6,8-10]. Empowerment is imperative in musculoskeletal health care, underscoring efforts to reframe treatment as less about curing and more about self-management [7]. Multiple expert consensus statements [10-19] include patient education in their clinical guidelines, and further research on patient education is needed for some clinical areas [14,15,20,21].

Multimedia, by definition, is the combination of images and words and has been used to increase learning and understanding since 1657, when the first children’s picture book, *Orbis Pictus*, was created, to the current day, when numerous digital multimedia platforms permeate life [22]. This is also true in health care, where multimedia patient education materials (PEMs) combine images and words in an effort to increase patient learning and understanding. It may be more advantageous to provide PEMs in multimedia format [23,24], such as leaflets, posters, infographics, or videos, than in traditional text-only format or verbal, face-to-face format, which can be burdensome in certain clinical settings [25], understaffed health care systems [26], or rural and remote locations without direct access to desired clinical care [27,28]. Traditionally, PEMs in musculoskeletal health care relied on printed or film formats, and while these materials can be effective, they lack the engagement and interactivity offered by digital educational interventions, leveraging multimedia elements such as videography, animations, interactive websites, and mobile apps to enhance patient education. The advantage of using such PEMs is that, once developed, the burden of delivery is very low when they can be disseminated cheaply, en masse [26,29,30], and without physical proximity [31,32]. Condensing the findings of health care research into these consumable formats with wide dispersal potential is particularly helpful for emerging health care systems in underresourced countries where face-to-face encounters are not always feasible [26]. A proposed disadvantage of PEMs is that they are generally not individualized to the patient [30], but this can be overcome by modern educational interventions possessing the digital capacity to tailor themselves to the user [33] or allow the addition of remote support [34]. Tracking the sharing of or engagement with such PEMs may help ensure that new, innovative metrics are used to translate research into practice as opposed to traditional citations [35].

Multimedia education research has seen a series of multimedia learning principles emerge based on empirical studies on how to maximize engagement and learning. One prominent example is the cognitive theory of multimedia learning (CTML) proposed by Mayer [22], which outlines 15 principles according to which educational multimedia should be designed to maximize learning and engagement. This theory suggests that learning is more effective when information is presented through multiple channels (eg, visual and auditory) using spoken words alongside images and in a manner that reduces cognitive overload. For example, the “segmenting principle” states that materials should be split into shorter, user-paced chunks, while the “signaling principle” recommends the use of text or symbols to highlight important information. Since its original publication in 2005 [36], a catalog of research has independently replicated and verified each of the 15 principles from the CTML [22]. This provides an opportunity to optimize the design of PEMs, given that many previous frameworks and scientific advice have focused on different aspects of optimizing the educational content [37-39]. Furthermore, the CTML framework has been applied to health research, where it has informed the design of health care education materials provided to practitioners [40-42], students [43-47], and patients with nonmusculoskeletal conditions [48-53]. It has the potential to inform studies of patients with musculoskeletal conditions as well. One study of low back pain videos found no strong correlates between user engagement and location or setting, duration, conflict of interest risk, speaker’s professional designation, source of the video, or clinical recommendations but did recommend that future research should focus on more detailed analyses of audiovisual aspects that may affect engagement [54]. This demonstrates a gap in the education research of patients with musculoskeletal conditions, where the CTML could be useful in correlating design features with engagement. Given the newfound ease in rapidly creating multimedia video content, the new digital age could benefit from its theories.

The dissemination of multimedia content is more effective if it is engaging and is more likely to be watched by more people for a longer duration [55]. The engagement of people with educational content results from more than simply presenting them with scientifically rigorous findings. Patients are now digital citizens [56,57] who must ration their attention across a spectrum of multimedia information, where science competes with misinformation [58-60], especially true in musculoskeletal health care [54,61-64]. Health care researchers and providers must keep their PEMs scientifically current and accurate [65], but they cannot rely on the content alone to sell the PEMs, if they fail to optimize engagement. Such shortcomings are more likely when the research of PEMs lack sufficient description and reporting standards [25,66]. Difficulty was noted when trying to retrieve and examine PEMs used in low back pain research [66], so this should be confirmed in the wider musculoskeletal literature. This may also reiterate the need to continually promote open science so that patient education interventions are available for appraisal and replication studies.

Research often focuses on the scientific content of PEMs rather than the design characteristics that promote knowledge transfer [67], but there has been limited design advice published in the musculoskeletal field, typically narrative advice from rehabilitation-based journals or authors. These commonly include the concise use of text and images in close proximation while avoiding redundancy between them [68], limitations on the amount of color but still using color to hasten the highlighting of pertinent information [68,69], or limitations on word count [70], to name a few. This provides a starting point for ensuring that the plethora of musculoskeletal guidelines promoting patient education are delivered in the most effective manner.

With further exploration into this area of patient education, there may be optimal strategies that inform the design of multimedia PEMs and draw attention away from inaccurate musculoskeletal health care messaging. Examining how their design is reported and described could aid in future trials.

### Objectives

The objectives of this review were as follows: (1) to identify randomized controlled trials (RCTs) in the area of adult musculoskeletal health care that used multimedia PEMs as a treatment or component of a treatment and compared them to any other interventions; (2) to examine how these interventions were reported with respect to their reproducibility and appraisability; and (3) to identify whether common design characteristics, such as digital versus nondigital format or adherence to CTML principles, were reported as affecting effectiveness.

## Methods

This systematic review was prepared according to the PRISMA (Preferred Reporting Items for Systematic Reviews and Meta-Analyses) [71] Multimedia Appendix 1) and PERSiST (PRISMA in Exercise, Rehabilitation, Sport Medicine and Sports Science) [72] guidelines. It was prospectively registered with the PROSPERO (CRD42022292134).

### Information Sources

PubMed, CINAHL, PsycINFO, and Embase were searched from inception to November 26, 2021. An updated search was carried out on August 10, 2022, and again on September 20, 2023, to identify any new potential studies.

### Eligibility Criteria

The population, intervention, comparison, outcomes, and study design (PICOS) framework was used to specify the eligibility criteria for this systematic review. The review sought RCTs of those aged ≥18 years with musculoskeletal conditions, defined by the World Health Organization as any condition of the joints, bones, muscles, or multiple body areas and systems that leads to temporary or lifelong limitations in function and participation [73]. Studies were included if they used any multimedia-based education intervention and examined it against any comparator. Multimedia-based educational interventions included any combination of reusable words and images that was delivered to patients. Examples included infographics, books, pamphlets, and videos. Studies were to include a knowledge outcome (primary outcome measure) and any patient-reported outcomes, including pain, disability, or self-efficacy (secondary outcome measure). No restrictions were applied to the follow-up periods or number of time points during which each outcome measure was obtained.

Exclusion criteria consisted of populations with nonmusculoskeletal conditions and the use of educational interventions that relied on clinician-delivered education with no provision of materials.

### Search Strategy

A detailed search strategy combined key concepts of the PICOS framework, such as “instruction” (including “patient education,” “information,” and “home exercise programme”), “multimedia” (including “video,” “audiovisual,” and “mobile device”), and “traditional format” (including “written,” “brochure,” and “information sheet”). Individual keywords and Medical Subject Headings terms for each concept were first combined with “OR” and then combined with the “AND” operator. No date or language restrictions were applied. Studies to be screened for inclusion were drawn from this search, and backward reference search was conducted among the included studies. Relevant gray literature was also searched. The detailed search strategy was registered on PROSPERO [74] and is available in Multimedia Appendix 2.

### Selection Process

Titles and abstract screening was conducted by all 6 authors using Covidence (Veritas Health Innovation) [75], and articles were advanced to full-text review when 2 authors agreed. Disagreements were resolved through consensus between the primary (GVO) and supervising (CD) authors.

Full-text review was conducted independently by the primary (GVO) and supervising (CD) authors, and all articles upon which an agreement was reached were advanced to the data extraction phase. Finally, a screening of the reference lists of all included studies was performed by the primary author (GVO), and any studies meeting inclusion were added. Any conflicts throughout this process were resolved through consensus between the primary (GVO) and supervising (CD) authors.

### Data Collection

Data extraction was conducted by the primary author (GVO), who then cross-referenced these findings with those from a second data extraction process conducted by 4 other authors (AP, CD, KM, and KB). Conflicts were resolved through consensus between the primary (GVO) and supervising (CD) authors.

In cases where an included study lacked sufficient detail about the PEMs used, a request for further information was emailed by the supervising author (CD). If no reply was received, then an additional request was sent 4 months later. If no reply was received within 1 month of this second attempt, the study was still eligible for inclusion, but the materials were coded as “irretrievable.”

### Data Items

The primary outcome was the retention of knowledge from the educational intervention, which was evaluated using, for example, a short answer test or a multiple-choice questionnaire. Any patient-reported outcomes were secondary outcomes. The PEMs that could be retrieved were evaluated according to the CTML principles. Variables of studies and participants were also recorded and included title, authors, author sex, year of publication, country of origin, musculoskeletal condition and population, outcome measures, sample size, age, study design, educational intervention, comparator intervention, and inclusion or retrievability of PEMs.

No assumptions were made in cases of missing data. In cases of multiple or lengthy PEMs being provided to an intervention arm, a sample was taken among all the materials to evaluate conformity with the CTML principles, as agreed by consensus between the primary (GVO) and supervising (CD) authors.

### Effect Measures

Where possible, effect sizes for sufficiently homogenous populations, interventions, and outcomes were combined so that an appropriate meta-analysis could be completed. A unitless measure of treatment effect size, such as standardized mean difference or Cohen *d*, was to be used.

### Synthesis Methods and Statistical Analysis

For the narrative synthesis, data regarding the components of the PEMs were extracted by the primary author (GVO), and cross-referenced against a second extraction that was performed by 4 other authors (AP, CD, KM, and KB). Conflicts were resolved through consensus between the primary (GVO) and supervising (CD) authors. Following the CTML principles proposed by Mayer [22], interventions were coded (yes, no, or not applicable) in a similar manner and synthesized based on the 15 design principles, as shown in Table 1.

**Table 1:** Explanation of the design principles from the cognitive theory of multimedia learning proposed by Mayer [22].

Design principle	Explanation
1. Multimedia principle	People learn better from words and pictures than from words alone.
2. Coherence principle	People learn better when extraneous material is excluded rather than included.
3. Signaling principle	People learn better when cues are added that highlight the organization of the essential material.
4. Redundancy principle	People do not learn better when printed text is added to graphics and narration. People learn better from graphics and narration than from graphics, narration, and printed text, when the lesson is fast paced.
5. Spatial contiguity principle	People learn better when corresponding words and pictures are presented near rather than far from each other on the page or screen. For example, in an animation on lightning formation, captions are presented at the bottom of the screen (separated presentation) or are placed next to the event they describe in the animation (integrated presentation).
6. Temporal contiguity principle	People learn better when corresponding words and pictures are presented simultaneously rather than successively. For example, the learner first views an animation on lightning formation and then hears the corresponding narration or vice versa (successive group), or the learner views an animation and hears the corresponding narration at the same time (simultaneous group).
7. Segmenting principle	People learn better when a multimedia message is presented in user-paced segments rather than as a continuous unit.
8. Pretraining principle	People learn more deeply from a multimedia message when they know the names and characteristics of the main concepts.
9. Modality principle	People learn more deeply from pictures and spoken words than from pictures and printed words.
10. Personalization principle	People learn better from multimedia presentations when words are in a conversational style rather than a formal style. For example, in a narrated animation on how the human lungs work, personalization involves using “you” and “your” in the narration script, such as “your nose” rather than “the nose” and “your throat” rather than “the throat.”
11. Voice principle	People learn better from multimedia presentations when words are spoken in an appealing human voice.
12. Image principle	People do not learn better from multimedia presentations when a static image of the instructor is added to the screen.
13. Embodiment principle	People learn more deeply from multimedia presentations when an onscreen instructor displays high embodiment rather than low embodiment.
14. Immersion principle	People do not necessarily learn better in 3D immersive virtual reality than with a corresponding 2D desktop presentation.
15. Generative activity principle	People learn better when they are guided in carrying out generative learning activities during learning (eg, summarizing, mapping, drawing, imagining, self-testing, self-explaining, teaching, or enacting). For example, after each of the 6 sections in a virtual reality simulation on the human bloodstream, students are asked to verbally summarize what they have learned.

### Reporting Bias Assessment

The Cochrane Risk of Bias-2 tool was used to review the bias of the included studies [76]. Two assessors independently evaluated the risk of bias in each of the included studies, and any interassessor disagreement was resolved through consensus between the primary (GVO) and supervising (CD) authors.

### Certainty Assessment

The Template for Intervention Description and Replication (TIDieR) checklist [77] was used to determine the quality of the RCTs that included their PEMs by 2 independent reviewers, with any conflicts resolved through consensus between the primary (GVO) and supervising (CD) authors.

## Results

### Study Selection

**Figure 1:**
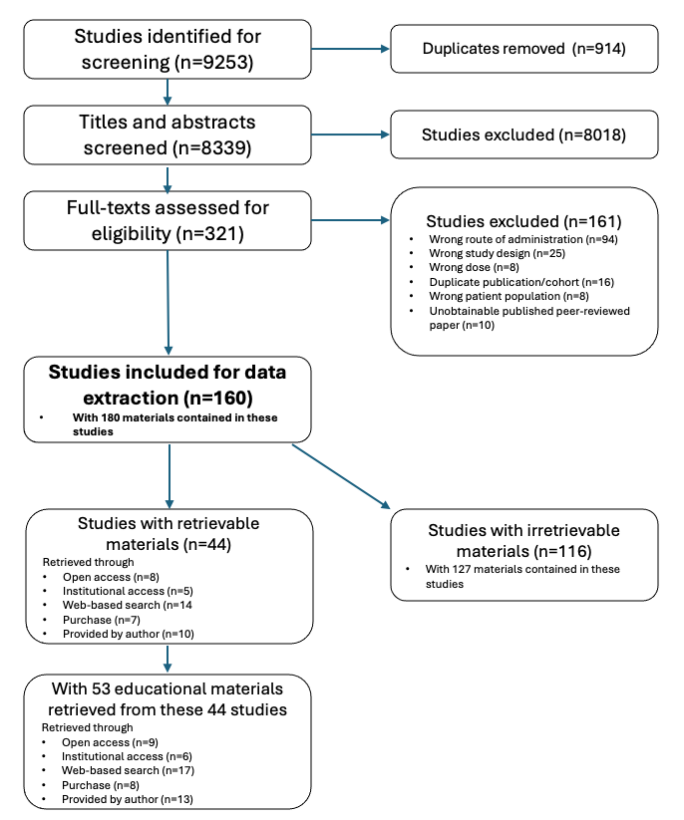
PRISMA (Preferred Reporting Items for Systematic Reviews and Meta-Analyses) flow diagram.

The PRISMA flow diagram in Figure 1 demonstrates the selection process.

Of the 176 studies originally deemed eligible for inclusion, 16 (9.1%) were based on patient cohorts used in previous publications, so these were merged with their previously reported trials, leaving a data set of 160 patient cohorts (female patients: n=29,903, 56%). Patient characteristics are shown in Table 2.

**Table 2:** Characteristics of participants in included studies (N=29,903).

Characteristics					Values
**Sex, n (%)**					
		Male		12,293 (41.07)	
		Female		16,868 (56.36)	
		Other^a^		6 (0.02)	
		Not reported		736 (2.46)	
Age (y), range^b^					18-90
**Population or condition, n (%)**					
	**Spinal pain**				
			LBP^c^	12,963 (43.31)	
			Neck pain	2099 (7.01)	
			Back pain	908 (3.03)	
			WAD^d^	765 (2.56)	
			Spinal pain	290 (0.97)	
	**Radiculopathy**				
			LBP with or without radicular symptoms	729 (2.44)	
			Cervical or lumbar radiculopathy	67 (0.22)	
	**Pain conditions**				
			Chronic pain	5964 (19.9)	
			Fibromyalgia	1272 (4.3)	
			General pain	95 (0.3)	
	**All other conditions**				
			Osteoarthritis	1663 (5.56)	
			Knee pain	395 (1.32)	
			LE^e^	303 (1.01)	
			Tendinopathy	270 (0.9)	
			UE^f^	259 (0.86)	
			Sedentary	249 (0.83)	
			Rheumatoid arthritis	143 (0.48)	
			Migraine	116 (0.39)	
			TMJ^g^	86 (0.29)	
			Pelvic pain	82 (0.27)	
			Shoulder	38 (0.13)	
			Multiple conditions, injuries, or body regions	1064 (3.55)	

^a^This category was not defined in the 2 studies where it emerged.

^b^Range is given due to the heterogenous reporting of age.

^c^LBP: low back pain.

^d^WAD: whiplash-associated disorder.

^e^LE: lower extremity.

^f^UE: upper extremity.

^g^TMJ: temporomandibular joint.

The 160 included studies were conducted between 1995 and 2023, with 68% (n=108) published since 2016, when >10 publications per year began occurring more regularly. Female names accounted for 72 (54%) of the 160 primary authors. Most studies originated from the United States (38/160, 23.8%), followed by Spain (20/160, 12.5%), Germany (12/160, 7.5%), Australia (10/160, 6.3%), and European Union countries (73/160, 45.6%). According to World Bank definitions [78], of the 160 studies, 137 (85.6%) came from high-income countries, followed by 19 (11.9%) from upper middle–income countries and 4 (2.5%) from lower middle–income countries. Further data on the country of origin are available in Multimedia Appendix 3.

### Risk of Bias

Appraisal of the included studies using Risk of Bias-2 found a high risk of bias in ≥2 of the 6 domains in 31 (19.4%) of the 160 studies, while the remaining 129 (80.6%) studies had a high risk of bias in none or just 1 of the domains. The full results can be found in Multimedia Appendix 3.

### Results of Individual Studies

The full findings of the 160 included studies can be found in Multimedia Appendix 3. In summary, the 160 studies used 180 multimedia PEMs in a variety of formats. A total of 99 (61.9%) studies incorporated digital delivery, with 57 (35.6%) using digital-only delivery and 41 (25.6%) using a combined digital and nondigital delivery. Another 57 (35.6%) studies used print-only delivery, and 4 (2.5%) used film-based delivery.

Of the 180 materials used across all the studies, the most commonly used materials were leaflets or pamphlets (n=67, 37.2%), followed by videos (n=37, 20.5%, of which 33, 18.3% were digital), combinations of all types of materials (n=31, 17.2%), websites (n=27, 15%), apps (n=18, 10%), manuals or workbooks (n=18, 10%), books (n=11, 6.1%), and presentation slides (n=7, 3.9%).

**Figure 2:**
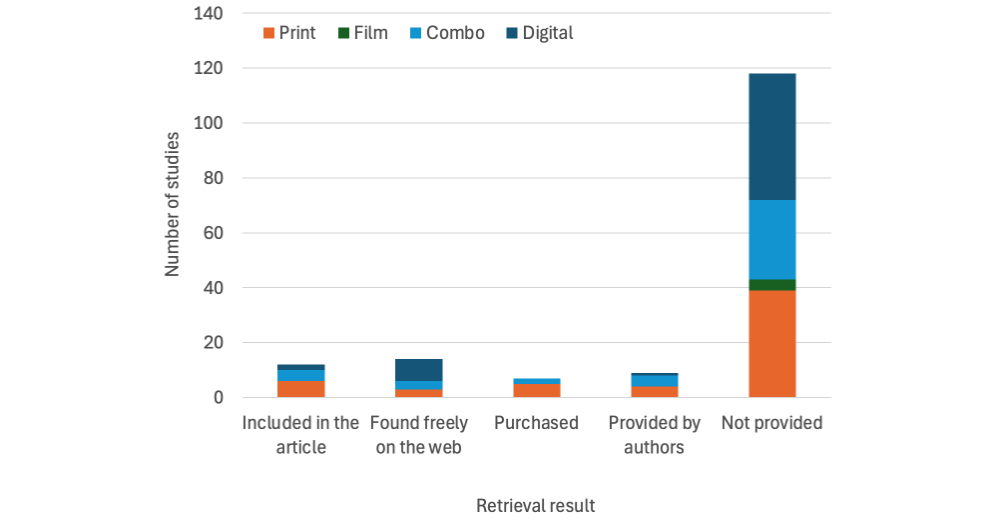
Types of materials for each retrieval method.

**Figure 3:**
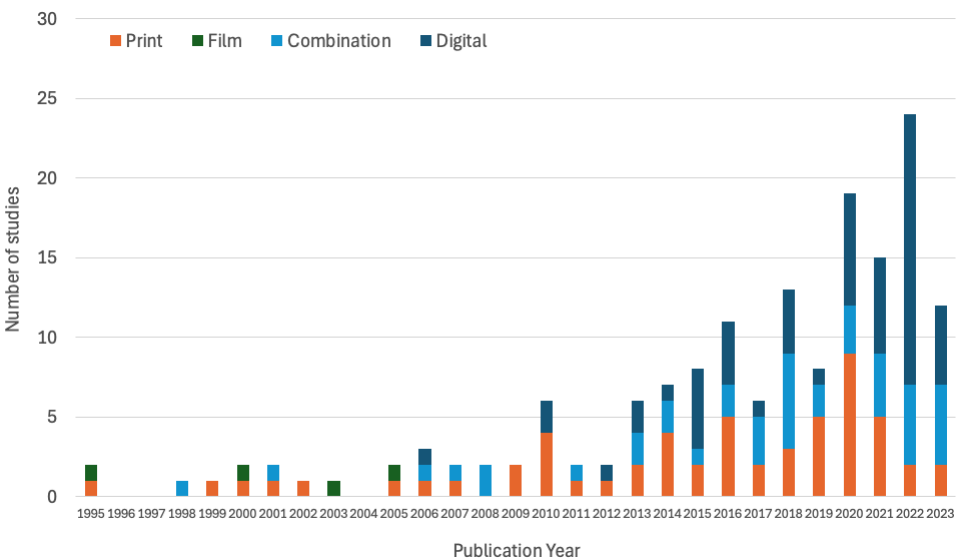
Types of educational materials used in musculoskeletal studies per year.

Of the 160 studies, 12 (7.5%) had materials that could be retrieved via their publication, and the materials of 30 (18.8%) studies were retrieved via a web search, purchase, or by request to the authors, who were contacted initially in April 2022 and again in August 2022 and May 2024 to request their materials. Overall, 44 (27.5%) different studies [30,79-120] provided 51 different PEMs for appraisal. Figure 2 shows the retrievability of the multimedia PEMs based on the type of delivery, and Figure 3 shows the retrievability based on the year of publication. Notably, materials requiring purchase were mostly books (average cost=11.55 EUR [US $14.54] per unit) or apps (average cost=7.50 EUR [US $8.07] per unit). 116 studies [28,29,32,58,121-232] contained materials that could not be retrieved.

The 51 multimedia PEMs that were retrieved were appraised according to the CTML principles (Table 3). When applicable, nearly all interventions adhered to the principles of immersion (44/46, 96%) by avoiding virtual reality, spatial contiguity (31/51, 94%) by displaying text and graphics in close proximity, voice 93% (14/15) by using an appealing human voice, temporal contiguity (46/50, 92%) by presenting text and graphics simultaneously, and personalization (42/48, 88%) by using words in a conversational style. Most interventions adhered to the segmenting principle (41/50, 82%) by presenting educational material in shorter segments instead of continuously, the signaling principle (37/51, 73%) by using cues to organize the information, the embodiment principle (7/10, 70%) by displaying the speaker, the pretraining principle (36/51, 71%) by familiarizing participants with main concepts in advance, and the image principle (8/17, 57%) by avoiding static images of speakers on screen.

**Table 3:** Appraisal of the design of multimedia educational materials using the cognitive theory of multimedia learning principles proposed by Mayer [22]^a^.

Study	Description of the educational intervention	Type	1	2	3	4	5	6	7	8	9	10	11	12	13	14	15
Bandak et al [79], 2022	Good Life with Osteoarthritis in Denmark (GLAD) education video [233]	Video (or film)	✓			✓	—^b^	✓		✓	✓	✓	✓	✓	✓	✓	
Baumeister et al [80], 2015	Video for a web psychological pain interventions	Video (or film)	✓	—		—	✓	—		✓	✓	✓	✓	✓	✓	✓	
Bennell et al [81], 2017	Website and videos [234]	Website or blog	✓	✓	✓		✓	✓	✓		✓	✓	✓	✓		✓	
Berberoğlu and Ülger [82], 2023	Multimedia instructions for motor control exercises (videos) [235]	Video (or film)	✓	✓	✓	✓	—		✓	✓	✓	✓	✓		—	✓	
Berberoğlu and Ülger [82], 2023	Face-to-face instructions for motor control exercises and handouts	Leaflet, pamphlet, or booklet	✓	✓	✓	✓	✓	✓	✓	✓	—		—	—	—	—	
Bostrøm et al [83], 2023	EPIO app	App	✓		✓		✓		✓	✓	✓	✓	✓		—	✓	✓
Chenot et al [84], 2019	German version of The Back Book (Rückenbuch)	Book	✓				✓	✓	✓			✓	—	—	—	✓	
Chimenti et al [85], 2023	PSE^c^ and exercise: videos and handouts	Multiple: videos and leaflets	✓	✓	✓		✓	✓	✓	✓	✓	✓	✓	✓	✓	✓	✓
Coudeyre et al [86], 2006	French version of The Back Book (Le Guide du Dos)	Book	✓				✓	✓	✓			✓	—	—	—	✓	
Coudeyre et al [87], 2007	French version of The Back Book (Le Guide du Dos)	Book	✓				✓	✓	✓			✓	—	—	—	✓	
Cramer et al [88], 2013	Written yoga instructions	Leaflet, pamphlet, or booklet	✓				✓	✓	✓	✓			—	—	—	✓	
Cramer et al [88], 2013	Self-care manual for neck pain and stiffness	Manual or workbook	✓		✓		✓	✓	✓	✓		✓	—	—	—	✓	
Dobscha et al [90], 2008	APT^d^ manual and worksheet	Manual or workbook	✓		✓		✓	✓	✓	✓		✓	—	—	—	✓	✓
Gardner et al [91], 2019	Participant handbook	Manual or workbook	✓				✓	✓		✓		✓	—	—	—	✓	✓
George et al [92], 2009	The Back Book	Book	✓				✓	✓	✓			✓	—	—	—	✓	
Gibbs et al [93], 2022	Pain education TEDx video: [236]	Video (or film)	✓	✓		✓	—	✓		✓	✓	✓	✓	✓	✓	✓	
Hrkać et al [94], 2022	Pictorial and descriptive examples of the exercise	Leaflet, pamphlet, or booklet	✓				✓	✓	✓	✓	—	—	—	—	—	✓	
Ibrahim et al [95], 2023	Booklet containing key information about the program	Leaflet, pamphlet, or booklet	✓	✓	✓	✓	✓	✓	✓	✓	—		—	—	—	—	
Janevic et al [96], 2022	Positive Steps website with videos: [237]	Multiple types of media	✓		✓		✓	✓	✓	✓	✓	✓	✓	✓	✓	✓	✓
Jinnouchi et al [97], 2023	Brief-See (100 minute long and therapist delivered) and material-based education	Book	✓		✓	✓	✓	✓	✓	✓	—		—	—	—	—	✓
Kohns et al [98], 2020	Pain psychology and neuroscience video: [238]	Video (or film)	✓				✓	✓	✓		✓	✓	✓		—	✓	
Kohns et al [98], 2020	Four Rules for a Healthy Lifestyle: [239]	Video (or film)	✓	✓	✓	✓	✓				✓		—		—	✓	
Lamb et al [99], 2010	The Back Book	Book	✓				✓	✓	✓			✓	—	—	—	✓	e
Meeus et al [100], 2010	Illustrations taken from “Explain Pain”	Manual or workbook	✓		✓		✓	✓	✓	✓		✓	—	—	—	✓	
Mukhtar et al [101], 2022	Standard PNE^e^ and CSPNE^f^: slides, leaflet, and audio	Multiple types of media used	✓	✓	✓	✓	✓	✓	✓	✓	✓	✓	✓		✓	✓	✓
O’Keeffe et al [30], 2020	Cognitive Functional Therapy written information	Leaflet, pamphlet, or booklet	✓		✓		✓	✓	✓	✓	✓	✓	—	—	—	✓	✓
Pacella-LaBarbara et al [102], 2020	PTSD Coach app	App	✓		✓		✓	✓	✓	✓	✓	✓	✓	✓		✓	✓
Roseen et al [103], 2023	12 weekly Hatha yoga classes with videos: [240]	Video (or film)	✓	✓		✓		✓		✓	✓	✓	✓	✓	✓	✓	✓
Roseen et al [103], 2023	Home manual	Manual or workbook	✓		✓		✓	✓	✓	✓	—	—	—	—	—	—	✓
Roseen et al [103], 2023	Education using “The Back Pain Helpbook”	Book	✓		✓		✓	✓	✓			✓	—	—	—	✓	
Sandhu et al [104], 2023	MyOpoidManager booklet	Manual or workbook	✓		✓		✓	✓	✓	✓		✓	—	—	—	✓	✓
Sandhu et al [104], 2023	MyOpoidManager app	App	✓		✓			✓	✓	✓		✓	—	—	—	—	✓
Saper et al [105], 2017	The Back Pain Helpbook	Book	✓		✓		✓	✓	✓			✓	—	—	—	✓	
Sherman et al [107], 2005	The Back Pain Helpbook	Book	✓		✓		✓	✓	✓			✓	—	—	—	✓	✓
Sherman et al [108], 2011	The Back Pain Helpbook	Book	✓		✓		✓	✓	✓			✓	—	—	—	✓	✓
Simula et al [109], 2021	Booklet [241]	Leaflet, pamphlet, or booklet	✓		✓		✓	✓		✓		✓	—				
Singh et al [110], 2018	Written instructions for opioid medication use and disposal	Leaflet, pamphlet, or booklet	✓		✓		✓	✓	—			✓	—	—	—	✓	
Skou et al [111], 2015	PowerPoint slides on exercise, education, diet, insoles and pain medication treatment presentation slides	PowerPoint (Microsoft Corporation) slides	✓	✓	✓		✓	✓	✓	✓		✓	—	—	—	✓	
Skou et al [111], 2015	Written information on knee osteoarthritis	Leaflet, pamphlet, or booklet	✓		✓		✓	✓	✓			✓	—	—	—	✓	
Skou et al [111], 2015	Written information on treatment and healthy lifestyle	Leaflet, pamphlet, or booklet	✓		✓		✓	✓	✓			✓	—	—	—	✓	
Syed et al [112], 2018	Narrated video on risks of narcotic overuse and abuse	Video (or film)	✓		✓		✓		✓	✓	✓	✓	✓		—	✓	
Thompson et al [113], 2016	Written information on serious nerve pathology and chronic cycle of pain	Leaflet, pamphlet, or booklet	✓		✓	✓	✓	✓	✓	✓		✓	—	—	—	✓	✓
Thorn et al [114], 2018	Pain education workbook	Manual or workbook	✓		✓		✓	✓	✓	✓		✓	—	—	—	✓	✓
Traeger et al [115], 2019	Intensive patient education	Manual or workbook	✓	✓	✓	✓	✓	✓	✓	✓			—	—	—		
Valiente-Castril lo et al [116], 2021	Chronic pain video: [242]	Video (or film)	✓	✓			✓	✓		✓	✓	✓	✓		—	✓	
Vanti et al [117], 2019	User manual	Manual or workbook	✓		✓		✓	✓	✓	✓		✓	—	—	—	✓	
Vanti et al [117], 2019	Informative brochure	Leaflet, pamphlet, or booklet	✓	✓	✓	✓	✓	✓	✓	✓		✓	—	—	—	✓	
Walsh et al [118], 2020	Supporting handbook and supplementary patient booklet	Multiple (see description)	✓		✓		✓	✓		✓		✓	—	—	—	✓	✓
Westenberg et al [119], 2018	Mindfulness-based video exercise: [243]	Website or blog	✓	✓	✓	✓		✓	✓	✓		✓	—	—	—	✓	✓
Yuan et al [120], 2021	Traditional paper book consisting of 64 pages	Book	✓	—	✓	—	✓	✓	✓	✓		—	—	—	—	✓	✓

^a^Links are included where materials are found freely on the web. Relevant information included for materials requiring online search or purchase.

^b^Not applicable due to the nature of educational materials or due to the inability to translate the language of materials.

^c^PSE: patient science education.

^d^APT: assistance with patient treatment.

^e^PNE: pain neuroscience education.

^f^CSPNE: culturally sensitive pain neuroscience education.

With respect to the principles with the poorest adherence, a minority of interventions adhered to the remaining principles of generative activity (21/51, 41%) by including any generative learning activities for the learner to carry out, modality (16/46, 35%) by opting for pictures accompanied by spoken words over written words, coherence (14/49, 29%) by excluding extraneous information, and redundancy (13/49, 27%) by avoiding redundant text alongside graphics.

The interrater agreement between the authors conducting the CTML appraisal was 87% on initial scoring and then 100% after any conflicts were discussed and consensus was reached between the primary (GVO) and supervising (CD) authors.

### Outcome Measures

Of the 160 included studies, 5 (3.1%) studies reported on the primary outcome for this review, knowledge translation or retention. The heterogeneity of the participants across these 5 studies precluded the planned meta-analysis of the primary outcome.

As for the secondary outcome of any patient-reported measures, the most frequently reported measure was pain intensity (89/160, 55.6%), followed by the Pain Catastrophizing Scale (42/160, 26.2%), the Roland Morris Disability Questionnaire (29/160, 18.1%), the Oswestry Disability Index (26/160, 16.2%), the Tampa Scale for Kinesiophobia (25/160, 15.6%), the Neck Disability Index (23/160, 14.4%), and patient satisfaction (26/160, 16.2%).

### Certainty of the Reporting of Interventions

The TIDieR checklist is shown in Table 4 and reflects the checks performed on the 44 studies that provided at least a sample of their multimedia PEMs. The checklist items that were mostly commonly missing from the PEMs were the reporting of who delivered the intervention (16/44, 36% studies) and where the provision of the intervention took place (14/44, 32% studies).

**Table 4:** Template for Intervention Description and Replication (TIDieR) checklist [77].

	1	2	3 and 4			5		6		7		8		9		10		11 and 12	
Study	Brief name	Why	What			Who provided		How		Where		When and how much		Tailoring		Modifications		How well	
Bandak et al [79] 2022	✓	✓	✓	✓	✓		✓		✓		✓		✓		—^a^		—		—
Baumeister et al [80], 2015	✓	✓		✓			✓		✓						—				
Bennell et al [81], 2017	✓		✓	✓	✓		✓		✓		✓				—				✓
Chenot et al [84], 2019		✓		✓			✓		✓		✓		—		—				✓
Coudeyre et al [87], 2007	✓	✓	✓	✓			✓		✓				—		—		—		—
Coudeyre et al [86], 2006	✓	✓	✓		✓		✓		✓		✓		—		—		—		—
Cramer et al [88], 2013	✓			✓	✓		✓				✓		—		—				
Dobscha et al [90], 2008							✓		✓				—		—		✓		
Gardner et al [91], 2019	✓	✓	✓	✓	✓		✓				✓				—		—		—
George et al [92], 2009	✓	✓	✓	✓			✓		✓		✓		—		—		—		—
Gibbs et al [93], 2022	✓	✓	✓	✓	✓		✓		✓		✓		—		—		—		—
Janevic et al [96], 2022	✓	✓	✓	✓	✓		✓		✓		✓		✓		—				Y
Kohns et al [98], 2020	✓	✓	✓	✓									—		—		—		—
Lamb et al [99], 2010	✓	✓		✓			✓		✓		✓		—		—				✓
Meeus et al [100], 2010	✓	✓	✓				✓				✓		—		—		—		—
O’Keeffe et al [30], 2020	✓			✓			✓				✓		—		—		✓		
Saper et al [105], 2017	✓	✓	✓	✓			✓				✓		—		—				
Sherman et al [xx], 2011	✓	✓			✓		✓		✓		✓		—		—				✓
Sherman et al [107], 2005	✓	✓			✓		✓		✓		✓		—		—		—		—
Simula et al [109], 2021	✓	✓	✓	✓	✓		✓		✓				—		—		—		—
Singh et al [110], 2018	✓		✓	✓									—		—		—		—
Skou et al [111], 2015		✓	✓	✓			✓				✓		✓		—		—		—
Syed et al [112], 2018	✓		✓	✓			✓						—		—		—		—
Thompson et al [113], 2016	✓	✓	✓	✓	✓		✓						—		—				✓
Thorn et al [114], 2018	✓	✓	✓	✓	✓		✓				✓		—		—		✓		✓
Traeger et al [115], 2019	✓	✓	✓	✓	✓		✓		✓		✓		✓		✓		✓		✓
Valiente-Castrillo et al [116], 2021	✓	✓	✓	✓	✓		✓				✓		—		—		—		—
Vanti et al [117], 2019	✓	✓	✓	✓							✓		—		—		—		—
Walsh et al [118], 2020	✓		✓				✓		✓		✓		✓		✓		N		✓
Westenberg et al [119], 2018	✓	✓		✓			✓		✓		✓		✓		—		—		—
Pacella-LaBarbara et al [102], 2020	✓	✓	✓	✓	✓		✓		✓		✓						✓		✓
Roseen et al [103], 2023	✓	✓	✓	✓	✓		✓		✓		✓		✓				✓		✓
Sandhu et al [104], 2023	✓	✓	✓	✓	✓		✓		✓		✓		✓		✓		✓		✓
Mukhtar et al [101], 2022	✓	✓	✓	✓	✓		✓		✓		✓		✓				✓		✓
Jinnouchi et al [97], 2023	✓	✓	✓	✓	✓		✓		✓		✓		✓				✓		✓
Hrkać et al [94], 2022	✓	✓	✓	✓	✓		✓		✓		✓		✓				✓		✓
Ibrahim et al [95], 2023	✓	✓	✓	✓	✓		✓		✓		✓		✓				✓		✓
Diab et al [89], 2022	✓	✓	✓	✓	✓		✓		✓		✓				✓		✓		✓
Chimenti et al [85], 2023	✓	✓	✓	✓	✓		✓		✓		✓		✓		✓		✓		✓
Berberoğlu and Ülger [82], 2023	✓	✓	✓	✓	✓		✓		✓		✓		✓				✓		✓
Bostrøm et al [83], 2023	✓	✓	✓	✓	✓		✓		✓		✓		✓				✓		✓
Yuan et al [120], 2021	✓	✓	✓	✓	✓		✓						—		—				

^a^Not applicable.

## Discussion

### Principal Findings

The aims of this systematic review were to identify all musculoskeletal-related RCTs that delivered multimedia-based educational materials to patients, to evaluate the design characteristics of these materials, and to ascertain whether a relationship exists between their design and improvements in the patients’ knowledge or clinical outcomes. Unfortunately, not all of these aims could be achieved. Patient knowledge was rarely tested, and it was never tested in studies that provided their PEMs. Overall, of the 160 studies, 44 (27.5%) provided 51 PEMs that were synthesized as part of this review. Meta-analysis was not possible due to the low number of publications for which educational materials could be retrieved and due to the heterogeneity of outcomes and populations among those that were retrievable.

Of the 160 studies, multimedia PEMs could be initially accessed only for 26 (16.2%): 12 (7.5%) included their PEMs in the scientific report, while 14 (8.8%) used materials that were freely available on the web. Upon further efforts, materials were obtained through purchase for 7 (4.4%) studies, while the authors of 9 (5.6%) studies provided their educational materials upon direct contact from the supervising author (CD). The fact that 118 (73.8%) of the 160 studies failed to provide their multimedia PEMs or a means to access these materials is disappointing. It undermines the replicability of the research (much of which is publicly funded) and its potential to make a real-world impact in clinical practice. If clinicians are to use patient education as recommended across a plethora of clinical guidelines in musculoskeletal health care [10-19], then clinicians must be able to see and hear what the study participants saw and heard. Having direct access to the materials used in a study’s educational intervention (in the scientific article, as an appendix, or through the public domain, such as a website or social media channel) is vital to incorporating research findings into clinical practice. We noted, as shown in Figure 2, that the increasing use of digital interventions in musculoskeletal education has not improved this accessibility issue. For digital interventions to realize their full potential, it is crucial that researchers make their materials accessible, either through publication appendices or public repositories.

The inability to replicate and implement research protocols due to poor reporting has been justly criticized in prior musculoskeletal research related to exercise [244,245], biologics [246], or injury epidemiology [247]. Patient education must be held to a similar standard. Specific to the area of PEMs, it is possible that difficulties may arise due to issues around safeguarding intellectual property with potential commercial value. However, we contend that until the scientific community develops an understanding of how and why patients engage with and learn from multimedia-based educational materials and devises a series of design principles for specific PEMs (akin to the pedagogical research conducted by Mayer [22] among student populations), the pursuit of a commercial enterprise formed around a core intellectual property of PEMs is premature (Multimedia Appendix 3 [22,23,40,59,80,127,133]). Going forward, we would urge researchers in musculoskeletal health care to provide a means to access their educational content with a persistent identifier in the public domain.

Our analysis of the 160 RCTs included in this review shows that increasingly, a significant proportion of studies published since 2017 have incorporated digital formats, such as videos, websites, and mobile apps (Figure 3). This trend is quite possibly driven by advancements in technology, increased accessibility of digital devices, and potentially the remote health care solutions accelerated by the COVID-19 pandemic [28]. While printed handouts and physical materials are still present in the literature, the increasing proportion of digital formats used alone or with these physical materials underscores the importance of digital solutions in the future of musculoskeletal health care.

Indeed, because no design principles related to the design of multimedia-based educational materials exist for patient populations with musculoskeletal conditions, in this review, we evaluated the PEMs according to the 15 principles of the CTML proposed by Mayer [22]. While the CTML is not a framework explicitly designed for PEMs, the 15 principles described therein provided a mechanism to examine the design characteristics, having been used in nonmusculoskeletal [49-62] research and a previous musculoskeletal [121] study. All but 1 of the 51 sets of multimedia PEMs from the 44 appraised studies used at least half of the CTML principles in the design of their materials, and a third of the materials were found to use ≥75% of the principles. The CTML design principles that were mostly not adhered to were the coherence principle of excluding extraneous information, the redundancy principle of avoiding similar information conveyed via words and images, the modality principle of combining different senses (ie, visual and auditory), and the generative activity principle of participants engaging in an activity that recaps their learning. The practical upshot for researchers and clinicians seeking to design and develop engaging educational materials is that the design of these materials can easily be improved over the interventions examined in this review by including words and images that do not repeat each other, cutting as much extraneous information as possible, combining auditory information with visual information wherever possible, and including some form of interactive activity to recapitulate the material. These modifications can be made to many of the multimedia PEMs that are designed for patients with musculoskeletal conditions, whether in the form of websites, apps, or social media posts, and should form the basis for design recommendations of multimedia PEMs for patients with musculoskeletal conditions.

However, it is important to note that further research is required to validate these recommendations among patient cohorts, as the CTML was developed in third-level educational settings, and not in health care. Literature that has contributed to the discussion of PEMs to date has mainly focused on aspects surrounding content [248], delivery methods [77], and understandability [39] rather than design. While it may not be possible to standardize all educational resources according to their target population or demographic, large research bodies, including reputable academic journals, professional organizations, government bodies, and charitable organizations, are key stakeholders in maintaining scientific integrity in the design and reproducibility of their content. This is especially true as self-management and widespread remote delivery of PEMs to underresourced areas become increasingly important in the delivery of musculoskeletal care [7] and for increasing public knowledge.

Then, it was surprising that very few (5/160, 3.1%) of the studies included in this review evaluated knowledge transfer or knowledge retention, as the primary purpose of an educational intervention is a change in postintervention knowledge (ie, learning). This may undermine the validity of the 96.9% (155/160) of studies using other outcomes, as the relationship is not well understood between such outcomes and the outcome of knowledge transfer or retention, which should be used to evaluate patient education. If clinical guidelines are consistently recommending educating patients, then research practices should consistently evaluate the effectiveness of this education by examining an outcome related to learning. It has been noted in low back pain PEMs that knowledge is being underassessed [66], and our review found similar results. Disability, function, pain, or any other outcome is usually favored over knowledge when multimedia PEMs are used, as in 96.9% (155/160) of the included studies in this review. However, it can also be argued that testing knowledge retention or knowledge transfer may not matter, as some types of educational materials may be effective for reasons other than learning in the target cohort, but this can only be better explored if knowledge is routinely measured. Unlike the American College of Sports Medicine guidelines [249] that recommend various exercise interventions to different populations with musculoskeletal conditions, there is no equivalent framework for educational interventions in musculoskeletal health care. This can lead to significant variety among educational interventions in terms of their content, format, length, and method of delivery. The 160 studies included in this review demonstrated that variety even when the target population was the same, such as our finding of 41 low back pain studies using a huge variety of interventions and outcomes (Multimedia Appendix 3).

Even the best research can be distorted by poor design or thwarted by the superior design of misinformation. Put bluntly, science must be designed to be as appealing as pseudoscience and other competing interests when it comes to patient education [58,59]. Scientific information does not need to debate with or debunk misinformation, as has been shown in nonmultimedia PEMs for low back pain [250]. Scientific information simply needs to be presented in the most engaging way possible [55], and health care research can find that advice exists on how to maximize engagement with videos [251,252], especially in the era of highly influential social media platforms [253]. Such cross-disciplinary fertilization with public health research and social media engagement research would allow musculoskeletal researchers and clinicians to provide more effective education to patients by using basic strategies such as segmenting into shorter portions [251] or personalizing the narration and experience [253] as much as possible, as has been noted in the CTML principles [22].

### Limitations

There are several limitations to the articles included in this review. First, the increased number of studies on educational resources in the past decade, especially the last 5 years, reflects the broader surge in digital health care resources available to the public. It could be argued that studies of younger age groups, who are accustomed to more information resources being at their fingertips, may have different results from those of the studies included this review, which contained many middle-aged and older adults and did not separate younger age brackets.

Second and as previously mentioned, jurisdictions with underresourced or very remote health care systems may have a special interest in the design of multimedia PEMs, as they may be used as a frontline intervention when one-to-one clinical care is impossible at the population or community level. However, among the 160 studies in our review, only 4 (2.5%) studies were from lower middle–income countries, comprising only 1% (277/29,903) of the participants in this review, so most of this research appears to be biased toward populations from more resourced countries and not toward countries that may glean the most benefit.

In terms of methodological considerations, we were able to retrieve educational materials from only 44 (23.1%) of the 160 studies, so our findings about the commonly overlooked principles of coherence, redundancy, and generative learning may not be generalizable to the wider array of musculoskeletal research when more materials can be examined. In addition, the CTML has provided guidance for designing materials in various areas of health care education in the past [40-53], but this is an extrapolation of its original use for research into undergraduate university education. Patient education research lacks any comparable framework, and despite our best search efforts, most research on patient education resources focused on optimizing the educational content in terms of understandability and actionability [39] or in terms of literacy [38] but failed to capture an expansive number of potential design features. While the CTML was the most obvious guideline used in the literature to date, that does not prevent better frameworks developing in the future. Multimedia interventions pertaining specifically to health care require far more research to determine whether other frameworks could be more suitable, and we hope this review using the CTML serves as a launching point for such discussions.

### Implications and Future Recommendations

There is a significant gap between what social media companies and what health care researchers and practitioners know about engagement with their respective clientele, with the latter group not necessarily able to prioritize obtaining and using this skill set. Liaising with content creators to scientifically evaluate engagement holds huge potential in musculoskeletal health care. Harnessing even a portion of the engagement knowledge possessed by those involved in social media advertising, educating, or campaigning could prove very effective in disseminating musculoskeletal knowledge to patients. This requires liaising with a new discipline. In addition, research should focus on the impact of digital interventions on various patient outcomes and the mechanisms through which they influence learning and behavior change.

Another priority should be to achieve a higher standard of reporting in studies using educational interventions and to ensure that such studies always specify the medium of the interventions, such as graphic, video, or leaflet, and some form of quantifiable length, such as word count, length of time, or number of pages, especially in what should be rare instances when the actual materials cannot be provided to the reader. Research publication guidelines should reflect the obvious need for patient education interventions to be accurately and consistently described, as has been recommended for other interventions in musculoskeletal research, and publication guidelines should be influenced by the open science movement by providing the PEM interventions wherever possible. These recommendations also pertain to the appraisal and replication of such research, as supplying sufficient information is vital to accurate appraisal and replication. Notably, of the 160 studies included in this review, the 116 (72.5%) studies that failed to provide their educational materials would fail to fulfill the third item on the TIDieR checklist: “Materials: Describe any physical or informational materials used in the intervention, including those provided to participants or used in intervention delivery or in training of intervention providers” [77]. Such items need to be accurately reported in systematic reviews.

Studies should also ascertain whether patient knowledge was affected by measuring it as an outcome. As multimedia PEMs become increasingly digital and more accessible, this review provides a timely reminder that knowledge transfer and implementation science must be intertwined with musculoskeletal research to put research findings into practice.

Determining whether the research is different for a younger, more tech-savvy population is worthwhile. We intend to repeat this review in the pediatric population to determine whether differences exist [74].

### Conclusions

Multimedia PEMs are widely used in musculoskeletal health care but are not supplied or sufficiently described, as is expected of reporting in other musculoskeletal assessments or interventions in terms of appraisability or reproducibility. The expansion of digital PEMs has not addressed this issue. Patient education requires higher reporting standards so that its prescription can be better replicated, which means that multimedia PEMs must be retrievable for evaluation. While no studies in our small sample appear to fully optimize the design of their multimedia PEMs, there was a particular gap in trying to design materials that conform to the generative activity, modality, coherence, and redundancy principles of the CTML, but this could change if 27.5% (44/160) of studies on multimedia PEMs could provide their actual materials. Knowledge transfer and retention must be better assessed to better explore the mechanisms of patient education. These findings must be heeded to improve the delivery of education for patients musculoskeletal and create both better research and better clinical adoption in the face of competing interests from misinformation that exists within musculoskeletal health care.

## Appendix

Multimedia Appendix 1 PRISMA (Preferred Reporting Items for Systematic Reviews and Meta-Analyses) checklist.

Multimedia Appendix 2 Search strategy.

Multimedia Appendix 3 Participants’ country of origin, summary results, Risk of Bias-2 appraisal, and full reference list of the included studies.

## Abbreviations

CTML: cognitive theory of multimedia learning
PEM: patient education material
PERSiST: PRISMA in Exercise, Rehabilitation, Sport Medicine and Sports Science
PICOS: population, intervention, comparison, outcomes, and study design
PRISMA: Preferred Reporting Items for Systematic Reviews and Meta-Analyses
RCT: randomized controlled trial
TIDieR: Template for Intervention Description and Replication
