# Supplementary material for: An Evaluation of the Design of Multimedia Patient Education Materials in Musculoskeletal Health Care: Systematic Review
Source: JMIR Rehabil Assist Technol. 2024 Oct 15;11:e48154. doi: 10.2196/48154 (PMC11522670; doi:10.2196/48154)
Supplement: Multimedia Appendix 2 [file rehab_v11i1e48154_app2.pdf]

**Search Strategy**

| | |
|---|---|
| Pubmed | "Patient education" OR "health education" OR "health information" OR "instruction*" OR "home exercise program*" OR "information dissemination" OR "education of patient*" OR "Community Health Education" OR "Programmed Learning" OR "Information Distribution" OR "Information Sharing" OR "dissemination of information" OR ((((("Patient Education as Topic"[Mesh:NoExp]) OR "Health Education"[Mesh]) OR "Consumer Health Information"[Mesh]) OR "Programmed Instructions as Topic"[Mesh]) OR "Information Dissemination"[Mesh]) OR "Computer-Assisted Instruction"[Mesh] AND "Multimedia" OR "Video*" OR "Pamphlet*" OR "DVD" OR "MP3" OR "MP4" OR "smartphone Application*" OR "App" OR "Website*" OR "blog*" OR "weblog*" OR "Audio" OR "Internet" OR "Visual" OR "booklet" OR "leaflet" OR "Videorecording*" OR "video recording*" OR "video-recording*" OR "videotape*" OR "audiovisual" OR "audio-visual" OR "videodisc recording*" OR "handout*" OR "computer*" OR "Web-based" OR "brochure*" OR "smartphone*" OR "smart-phone*" OR "smart phone*" OR "iPad" OR "infographic*" OR "picture*" OR "mobile phone*" OR "mobile device*" OR "cell phone*" OR "MP3 Player*" OR "MP3-Player*" OR "i-POD*" OR "i POD" OR iPOD* OR "Mobile App*" OR "Portable Electronic App*" OR "Portable Software App*" OR "Instructional Films and Video" OR "Audio-Video" OR "podcast" OR "webcast" OR "Web-based Intervention*" OR "Online Intervention*" OR "consumer information handout" OR "Cellular Phone*" OR "Cellular Telephone*" OR "Mobile Phone*" OR (((((((((((((((((((("Multimedia"[Mesh]) OR "Videotape Recording"[Mesh]) OR "Video Recording"[Mesh]) OR "Videodisc Recording"[Mesh]) OR "Pamphlets"[Mesh]) OR "MP3-Player"[Mesh]) OR "Mobile Applications"[Mesh]) OR "Blog" [Publication Type]) OR "Video-Audio Media" [Publication Type]) OR "Audiovisual Aids"[Mesh]) OR "Instructional Film and Video" [Publication Type]) OR "Electronic Supplementary Materials" [Publication Type]) OR "Webcast" [Publication Type]) OR "Internet"[Mesh]) OR "Internet-Based Intervention"[Mesh]) OR "Patient Education Handout" [Publication Type]) OR "Computers"[Mesh:NoExp]) OR "Computers, Handheld"[Mesh]) OR "Computer-Assisted Instruction"[Mesh]) OR "Computer Graphics"[Mesh:NoExp]) OR "Smartphone"[Mesh]) OR "Motion Pictures"[Mesh]) OR "Cell Phone"[Mesh] AND "Musculoskeletal disease*" OR "orthopedic disorder*" OR "orthopaedic disorder*" OR "musculoskeletal patient*" OR "musculoskeletal pain*" OR "musculoskeletal injur*" OR orthopedic* OR orthopaedic* OR "Overuse Injur*" OR "Repetitive Motion Disorder*" OR "Repetitive Strain Injur*" OR "Overuse Syndrome*" OR "Repetitive Stress Injur*" OR "Cumulative Trauma Disorder" OR ((("Musculoskeletal Diseases"[Mesh]) OR "Musculoskeletal Pain"[Mesh]) OR "Orthopedics"[Mesh]) OR "Cumulative Trauma Disorders"[Mesh] AND "Chronic pain*" OR "Widespread Chronic Pain*" OR "long-term pain" OR "persistent pain" OR "constant pain" OR "recurring pain" OR "Recurrent pain" OR "continuous pain" OR "long- |

| | |
|---|---|
| | standing pain" OR "Life-long pain" OR ("Chronic Pain"[Mesh]) AND (((randomized controlled trial [pt] OR clinical trial, phase iii [pt] OR clinical trial, phase iv [pt] OR clinicaltrials.gov [si] OR isrctn [si] OR randomized controlled trials as topic [mh]) OR (clinical trial [pt] AND (((single [tw] OR double [tw] OR doubleblind [tw] OR doubleblinded [tw] OR treble [tw] OR triple [tw]) AND (blind [tw] OR blinded [tw] OR mask [tw] OR masked [tw] OR masks [tw] OR sham [tw] OR shams [tw] OR dummy [tw])) OR (random [tw] OR randomise [tw] OR randomize [tw] OR randomised [tw] OR randomized [tw] OR rct [tw] OR rcts [tw] OR single-blind method [mh] OR double-blind method [mh] OR random allocation [mh]))) AND ((comparative study [pt] OR compare [tw] OR compares [tw] OR compared [tw] OR comparing [tw] OR comparison [tw] OR comparative [tw] OR effective [tw] OR effectiveness [tw] OR versus [ti] OR vs [ti]) OR (activities of daily living [mh] OR benefit [tw] OR benefits [tw] OR budgets [mh] OR chronic disease [mh] OR clinical trials data monitoring committees [mh] OR cognitive function [tw] OR ec [sh] OR death [mh] OR diffusion of innovation [mh] OR discharge [tw] OR economics, pharmaceutical [mh] OR evidence based practice [mh] OR functional status [tw] OR guideline adherence [mh] OR harm [tw] OR harms [tw] OR health services research [mh] OR health status [mh] OR hospitalization [mh] OR interventions [tw] OR life expectancy [mh] OR longevity [mh] OR models, statistical [mh] OR models, theoretical [mh:noexp] OR morbidity [mh] OR mortality [mh] OR noninferior [tw] OR noninferiority [tw] OR outcome and process assessment [mh] OR outcome [tw] OR outcomes [tw] OR patient compliance [mh] OR postoperative care [mh] OR postoperative complications [mh] OR product surveillance, postmarketing [mh] OR propensity score [tw] OR quality-adjusted life years [mh] OR quality of life [mh] OR recovery of function [mh] OR recurrence [mh] OR relapse [tw] OR remission [tw] OR reoperation [mh] OR risk [tw] OR risk management [mh] OR survival analysis [mh] OR survival rate [mh] OR technology assessment, biomedical [mh] OR trial [ti] OR trials [ti]))) OR clinical effectiveness [tw]) NOT systematic [sb] |
| PsycINFO | (“Patient education” OR “health education” OR “health information” OR “instruction*” OR “home exercise program*” OR “information dissemination” OR “education of patient*” OR “Community Health Education” OR “Programmed Learning” OR “Information Distribution” OR “Information Sharing” OR “dissemination of information” OR MAINSUBJECT.EXACT("Client Education") OR MAINSUBJECT.EXACT("Consumer Education") OR MJMAINSUBJECT.EXACT("Health Education") OR MAINSUBJECT.EXACT("Digital Health Resources") OR MAINSUBJECT.EXACT("Health Information") OR MAINSUBJECT.EXACT("Information Dissemination") OR MAINSUBJECT.EXACT("Self-Management") OR MAINSUBJECT.EXACT("Programmed Instruction") OR |

| | |
|---|---|
| | MAINSUBJECT.EXACT("Information and Communication Technology") OR MAINSUBJECT.EXACT("Computer Assisted Instruction")) AND (“Multimedia” OR “Video*” OR “Pamphlet*” OR “DVD” OR “MP3” OR “MP4” OR “smartphone Application*” OR “App” OR “Website*” OR “blog*” OR “weblog*” OR “Audio” OR “Internet” OR “Visual” OR “booklet” OR “leaflet” OR “Videorecording*” OR “video recording*” OR “video-recording*” OR “videotape*” OR “audiovisual” OR “audio-visual” OR “videodisc recording*” OR “handout*” OR “computer*” OR “Web-based” OR “brochure*” OR “smartphone*” OR “smart-phone*” OR “smart phone*” OR “iPad” OR “infographic*” OR “picture*” OR “mobile phone*” OR “mobile device*” OR “cell phone*” OR “MP3 Player*” OR “MP3-Player*” OR “i-POD*” OR “i POD” OR iPOD* OR “Mobile App*” OR “Portable Electronic App*” OR “Portable Software App*” OR “Instructional Films and Video” OR “Audio-Video” OR “podcast” OR “webcast” OR “Web-based Intervention*” OR “Online Intervention*” OR “consumer information handout” OR “Cellular Phone*” OR “Cellular Telephone*” OR “Mobile Phone*” OR MAINSUBJECT.EXACT("Audiovisual Communications Media") OR MAINSUBJECT.EXACT("Multimedia") OR MAINSUBJECT.EXACT("Video-Based Interventions") OR MAINSUBJECT.EXACT("Computer Applications") OR MAINSUBJECT.EXACT("Mobile Applications") OR MAINSUBJECT.EXACT("Blog") OR MAINSUBJECT.EXACT("Websites") OR MJMAINSUBJECT.EXACT.EXPLODE("Instructional Media") OR MJMAINSUBJECT.EXACT("Educational Audiovisual Aids") OR MJMAINSUBJECT.EXACT("Internet") OR MJMAINSUBJECT.EXACT.EXPLODE("Video-Based Interventions") OR MJMAINSUBJECT.EXACT.EXPLODE("Videotape Instruction") OR MJMAINSUBJECT.EXACT.EXPLODE("Audiovisual Instruction") OR MJMAINSUBJECT.EXACT.EXPLODE("Digital Interventions") OR MAINSUBJECT.EXACT.EXPLODE("Mobile Phones") OR MAINSUBJECT.EXACT.EXPLODE("Mobile Devices") OR MAINSUBJECT.EXACT.EXPLODE("Smartphones") OR MJMAINSUBJECT.EXACT("Tablet Computers") OR MJMAINSUBJECT.EXACT("Mobile Applications") OR MJMAINSUBJECT.EXACT("Instructional Media") OR MAINSUBJECT.EXACT.EXPLODE("Digital Interventions") OR MJMAINSUBJECT.EXACT("Tablet Computers")) AND (“Musculoskeletal disease*” OR “orthopedic disorder*” OR “orthopaedic disorder*” OR “musculoskeletal patient*” OR “musculoskeletal pain*” OR “musculoskeletal injur*” OR orthopedic* OR orthopaedic* OR “Overuse Injur*” OR “Repetitive Motion Disorder*” OR “Repetitive Strain Injur*” OR “Overuse Syndrome*” OR “Repetitive Stress Injur*” OR “Cumulative Trauma Disorder” OR MJMAINSUBJECT.EXACT("Muscular Disorders") OR MJMAINSUBJECT.EXACT("Musculoskeletal Disorders”)) AND (“Chronic pain*” OR “Widespread Chronic Pain*” OR “long-term pain” OR “persistent pain” OR “constant pain” OR “recurring pain” OR “Recurrent pain” OR “continuous pain” OR “long-standing pain” OR “Life-long pain” |

| | |
|---|---|
| | OR MJMAINSUBJECT.EXACT("Chronic Pain") OR MJMAINSUBJECT.EXACT("Geriatric Patients")) AND (SU.EXACT("Treatment Effectiveness Evaluation") OR SU.EXACT.EXPLODE("Treatment Outcomes") OR SU.EXACT("Placebo") OR SU.EXACT("Followup Studies") OR placebo* OR random* OR "comparative stud*" OR clinical NEAR/3 trial* OR research NEAR/3 design OR evaluat* NEAR/3 stud* OR prospectiv* NEAR/3 stud* OR (singl* OR doubl* OR trebl* OR tripl*) NEAR/3 (blind* OR mask*) ) |
| Embase | ((('patient education' OR 'health education' OR 'health information' OR 'instruction*' OR 'home exercise program*' OR 'information dissemination' OR 'education of patient*' OR 'community health education' OR 'programmed learning' OR 'information distribution' OR 'information sharing' OR 'dissemination of information' OR 'patient education'/exp OR 'health education'/exp OR 'allied health education'/mj OR 'consumer health information'/mj OR 'information dissemination'/mj OR 'information distribution'/exp OR 'information sharing'/exp) AND 'multimedia' OR 'video*' OR 'pamphlet*' OR 'dvd' OR 'mp3' OR 'mp4' OR 'smartphone application*' OR 'app' OR 'website*' OR 'blog*' OR 'weblog*' OR 'audio' OR 'internet' OR 'visual' OR 'booklet' OR 'leaflet' OR 'videorecording*' OR 'video recording*' OR 'video-recording*' OR 'videotape*' OR 'audiovisual' OR 'audio-visual' OR 'videodisc recording*' OR 'handout*' OR 'computer*' OR 'web-based' OR 'brochure*' OR 'smartphone*' OR 'smart-phone*' OR 'smart phone*' OR 'ipad' OR 'infographic*' OR 'picture*' OR 'mobile device*' OR 'cell phone*' OR 'mp3 player*' OR 'mp3-player*' OR 'i-pod*' OR 'i pod' OR ipod* OR 'mobile app*' OR 'portable electronic app*' OR 'portable software app*' OR 'instructional films and video' OR 'audio-video' OR 'podcast' OR 'webcast' OR 'web-based intervention*' OR 'online intervention*' OR 'consumer information handout' OR 'cellular phone*' OR 'cellular telephone*' OR 'mobile phone*' OR 'multimedia'/mj OR 'videorecording'/mj OR 'publication'/mj OR 'mp3 player'/mj OR 'smartphone'/mj OR 'mobile application'/exp OR 'website'/mj OR 'blogging'/mj OR 'audio recording'/mj OR 'internet'/mj OR 'audiovisual aid'/mj OR 'web-based intervention'/mj OR 'tablet computer'/mj OR 'picture'/mj OR 'mobile phone'/mj OR 'mobile device'/mj OR 'ipod'/mj OR 'podcast'/mj OR 'webcast'/mj) AND 'musculoskeletal disease*' OR 'orthopedic disorder*' OR 'orthopaedic disorder*' OR 'musculoskeletal patient*' OR 'musculoskeletal pain*' OR 'musculoskeletal injur*' OR orthopedic* OR orthopaedic* OR 'overuse injur*' OR 'repetitive motion disorder*' OR 'repetitive strain injur*' OR 'overuse syndrome*' OR 'repetitive stress injur*' OR 'cumulative trauma disorder' OR 'musculoskeletal disease'/exp OR 'musculoskeletal pain'/mj OR 'musculoskeletal injury'/mj OR 'overuse injury'/mj OR 'cumulative trauma disorder'/exp OR 'joint injury'/mj OR 'sport injury'/mj) AND 'chronic pain*' OR 'widespread chronic pain*' OR 'long-term pain' OR 'persistent pain' OR 'constant pain' OR 'recurring pain' OR 'recurrent pain' OR 'continuous pain' OR 'long-standing pain' OR 'life-long pain' OR |

| | |
|---|---|
| | 'chronic pain'/mj AND 'double-blind*':ab,ti OR placebo*:ab,ti OR blind*:ab,ti |
| CINAHL | ‘Patient education’ OR ‘health education’ OR ‘health information’ OR ‘instruction*’ OR ‘home exercise program*’ OR ‘information dissemination’ OR ‘education of patient*’ OR ‘Community Health Education’ OR ‘Programmed Learning’ OR ‘Information Distribution’ OR ‘Information Sharing’ OR ‘dissemination of information’ OR (MM "Preoperative Education") OR (MM "Health Education") OR (MM "Patient Education") OR (MM "Patient Discharge Education") OR (MH "Health Education") OR (MH "Medical Illustration") OR (MH "Health Information") OR (MH "Consumer Health Information") OR (MH "Computer Assisted Instruction") OR (MH "Programmed Instruction") AND ‘Multimedia’ OR ‘Video*’ OR ‘Pamphlet*’ OR ‘DVD’ OR ‘MP3’ OR ‘MP4’ OR ‘smartphone Application*’ OR ‘App’ OR ‘Website*’ OR ‘blog*’ OR ‘weblog*’ OR ‘Audio’ OR ‘Internet’ OR ‘Visual’ OR ‘booklet’ OR ‘leaflet’ OR ‘Videorecording*’ OR ‘video recording*’ OR ‘video-recording*’ OR ‘videotape*’ OR ‘audiovisual’ OR ‘audio-visual’ OR ‘videodisc recording*’ OR ‘handout*’ OR ‘computer*’ OR ‘Web-based’ OR ‘brochure*’ OR ‘smartphone*’ OR ‘smart-phone*’ OR ‘smart phone*’ OR ‘iPad’ OR ‘infographic*’ OR ‘picture*’ OR ‘mobile phone*’ OR ‘mobile device*’ OR ‘cell phone*’ OR ‘MP3 Player*’ OR ‘MP3-Player*’ OR ‘i-POD*’ OR ‘i POD’ OR iPOD* OR ‘Mobile App*’ OR ‘Portable Electronic App*’ OR ‘Portable Software App*’ OR ‘Instructional Films and Video’ OR ‘Audio-Video’ OR ‘podcast’ OR ‘webcast’ OR ‘Web-based Intervention*’ OR ‘Online Intervention*’ OR ‘consumer information handout’ OR ‘Cellular Phone*’ OR ‘Cellular Telephone*’ OR ‘Mobile Phone*’ OR (MM "Multimedia") OR (MH "Videorecording") OR (MH "Pamphlets") OR (MH "Digital Versatile Disc") OR (MH "Videodiscs") OR (MH "Audiovisuals") OR (MH "Audiorecording") OR (MH "Posters") OR (MH "Mobile Applications") OR (MH "Smartphone") OR (MH "Cellular Phone") OR (MH "Internet-Based Intervention") OR (MH "Blogs") OR (MH "Informatics") OR (MH "Health Informatics") OR (MH "Computer Graphics") OR (MH "Computer Assisted Instruction") AND ‘Musculoskeletal disease*’ OR ‘orthopedic disorder*’ OR ‘orthopaedic disorder*’ OR ‘musculoskeletal patient*’ OR ‘musculoskeletal pain*’ OR ‘musculoskeletal injur*’ OR orthopedic* OR orthopaedic* OR ‘Overuse Injur*’ OR ‘Repetitive Motion Disorder*’ OR ‘Repetitive Strain Injur*’ OR ‘Overuse Syndrome*’ OR ‘Repetitive Stress Injur*’ OR ‘Cumulative Trauma Disorder’ OR (MH "Musculoskeletal Diseases+") OR (MH "Rehabilitation Patients") OR (MH "Muscle Pain") OR (MH "Back Pain") OR (MH "Groin Pain") OR (MH "Knee Pain") OR (MH "Neck Pain") OR (MH "Cumulative Trauma Disorders") AND ‘Chronic pain*’ OR ‘Widespread Chronic Pain*’ OR ‘long-term pain’ OR ‘persistent pain’ OR ‘constant pain’ OR ‘recurring pain’ OR ‘Recurrent pain’ OR ‘continuous pain’ OR ‘long-standing pain’ OR ‘Life-long pain’ OR (MH "Chronic Pain") AND (randomized controlled trials OR MH double-blind studies OR MH single-blind studies OR MH random assignment OR MH pretest-posttest design OR MH cluster sample OR TI (randomised OR randomized) OR AB |

| | |
|---|---|
| | (random*) OR TI (trial) OR (MH (sample size) AND AB (assigned OR allocated OR control)) OR MH (placebos) OR PT (randomized controlled trial) OR AB (control W5 group) OR MH (crossover design) OR MH (comparative studies) OR AB (cluster W3 RCT)) NOT ((MH animals+ OR MH animal studies OR TI animal model*) NOT MH human) |
