# Supplementary material for: An Evaluation of the Design of Multimedia Patient Education Materials in Musculoskeletal Health Care: Systematic Review
Source: JMIR Rehabil Assist Technol. 2024 Oct 15;11:e48154. doi: 10.2196/48154 (PMC11522670; doi:10.2196/48154)
Supplement: Multimedia Appendix 3 [file rehab_v11i1e48154_app3.docx]

**APPENDIX C: Country of origin**

| **Country** | **World Bank Income Category** | **Participants** | |
| --- | --- | --- | --- |
|  |  | **n** | **%** |
| USA | High | 10494 | 35.1% |
| Spain | High | 2112 | 7.1% |
| Germany | High | 2344 | 7.8% |
| Australia | High | 1598 | 5.3% |
| UK | High | 2440 | 8.2% |
| Brazil | Upper Middle | 815 | 2.7% |
| Canada | High | 825 | 2.8% |
| Sweden | High | 1202 | 4.0% |
| Denmark | High | 654 | 2.2% |
| Iran | Upper Middle | 415 | 1.4% |
| Hong Kong | High | 577 | 1.9% |
| Netherlands | High | 382 | 1.3% |
| Belgium | High | 303 | 1.0% |
| Ireland | High | 426 | 1.4% |
| Nigeria | Lower Middle | 203 | 0.7% |
| Saudi Arabia | High | 145 | 0.5% |
| France | High | 2479 | 8.3% |
| Switzerland | High | 153 | 0.5% |
| China | Upper Middle | 135 | 0.5% |
| Italy | High | 114 | 0.4% |
| Turkey | Upper Middle | 70 | 0.2% |
| Norway | High | 259 | 0.9% |
| Finland | High | 415 | 1.4% |
| Bosnia & Herzegovina | Upper Middle | 180 | 0.6% |
| Austria | High | 100 | 0.3% |
| South Africa | Upper Middle | 74 | 0.2% |
| Pakistan | Lower Middle | 50 | 0.2% |
| Japan | High | 46 | 0.2% |
| Korea | High | 43 | 0.1% |
| Thailand | Upper Middle | 42 | 0.1% |
| Jordan | Upper Middle | 41 | 0.1% |
| Croatia | High | 30 | 0.1% |
| Multi | High | 737 | 2.5% |
| ***Subtotal (EU)*** | *shaded regions* | *11389* | *38.1%* |
| ***Subtotal Income categories*** | *High Income* | *28065* | *94%* |
|  | *Upper Middle Income* | *1561* | *5%* |
|  | *Lower Middle Income* | *277* | *1%* |
| **TOTAL** | **ALL PARTICIPANTS** | 29903 | 100.0% |

**APPENDIX D - Summary of Results**

| **Author Year** | **DOI** | **Location** | **Injury/ Impairment** | **Total (N)** | **% female** | **Age: mean±SD or (range)** | **Intervention Education Type** | **Comparator Education Type** | **Method of delivery** | **Any part of materials retrievable? (Study n)** | |
| --- | --- | --- | --- | --- | --- | --- | --- | --- | --- | --- | --- |
| Aguirrezabal 2019 | <https://doi-org.ucd.idm.oclc.org/10.1017%2FS1463423619000720> | Vitoria-Gasteiz, Basque Country, Spain | Migraine | 116 | 82% |  | Multiple (see description) |  | Combo | | No |
| Alasfour 2022 | <https://www.tandfonline.com/action/showCitFormats?doi=10.1080/09638288.2020.1836268> | Saudi Arabia | Knee OA | 40 | 100% | 54.5±4.33 | App | Leaflet/Pamphlet/Booklet | Combo | | No |
| Albaladejo 2010 | 10.1097/BRS.0b013e3181b9c9a7 | Palma de Mallorca, Spain | LB±radicular pain | 348 | 67% |  | Leaflet/Pamphlet/Booklet | Leaflet/Pamphlet/Booklet | Print | | No |
| Almhdawi 2020 | <https://doi.org/10.1177/0269215520937757> | Jordan | cNSLBP | 41 | 54% | 40.5±7.2, 41.7±6.4 | App | Leaflet/Pamphlet/Booklet | Combo | | No |
| Amaral 2020 | 10.2519/jospt.2019.8839. | São Paulo, Brazil | cNSLBP | 222 | 75% | 47.9±15.5, 48.6±15.8 | Leaflet/Pamphlet/Booklet | Leaflet/Pamphlet/Booklet | Print | | No |
| Amer-Cuenca 2019 | https://doi.org/10.1093/pm/ pnz069 | Valencia, Alcorco􏰀n, Alcala􏰀 de Henares; Spain | Fibromyalgia | 77 | 92% |  | Multiple (see description) |  | Combo | | No |
| Archer 2016 | 10.1016/j.jpain.2015.09.013 | USA | undergoing a laminectomy ± arthrodesis for: lumbar degenerative condition (spinal stenosis, spondylosis ± myelopathy, and degenerative spondylolisthesis) multi | 86 | 56% | 57.6±12.2 | Manual/Workbook | Manual/Workbook | Print | | No |
| Areeudomwong 2016 | 10.1002/msc.1165 | Thailand | cNSLBP | 42 | 74% | 35.4±10.3, 36.2±9.9 |  | Leaflet/Pamphlet/Booklet | Print | | No |
| Ariza-Mateos 2020 | 10.1016/j.maturitas.2020.04.005 | Granada, Spain | chronic pelvic pain | 44 | 100% | 42.6±8.6, 45.35±9.5 |  | Leaflet/Pamphlet/Booklet | Print | | No |
| Bandak 2021 | 10.1136/ annrheumdis-2021-221129 | Copenhangen, Denmark | Knee OA | 206 | 46% | 70.1±8.3, 66.7±8.2 | Video (or film) |  | Digital | | Yes (included in open access article) |
| Barrenengoa-Cuadra 2021 | 10.1002/ejp.1738 | Basque country, Spain | fibromyalgia | 139 | 94% | 52.3±9.2, 51.4±10.2 | other (see description) |  | Combo | | No |
| Baumeister 2015 | 10.1097/AJP.0000000000000118 | Germany | chronic pain | 104 | 58% | 54.4±13.6 (18-84) | Video (or film) |  | Digital | | Yes (provided by authors on request) |
| Baumeister 2021 | 10.1159/000511881 | Uln, Germany | Chronic back pain | 209 | 60% | 49.9±9.36 | App |  | Digital | | No |
| Beltran-Alacreu 2015 | 10.1097/PHM.0000000000000293 | Madrid, Spain | cNSNP neck pain | 45 | 78% | 43.5±15.9, 40.8±16.2, 39.8±13.4 | Powerpoint slides | Leaflet/Pamphlet/Booklet | Print | | No |
| Bennell 2017 | 10.7326/M16-1714 | Melbourne, Australia | Knee pain | 148 | 56% | 60.8±6.5, 61.5±7.6 | Multiple (see description) | Website/Blog | Digital | | Yes (available online) |
| Berberoğlu 2023 | 10.1123/jsr.2022-0158 | Turkey | chronic NSLBP | 30 | #VALUE! | Face-to-face: Mean 40.7 years, SD 10.0; Multimedia: Mean 42.1 years, SD 9.2 | Video (or film) | Leaflet/Pamphlet/Booklet | Combo | | Yes (included in open access article) |
| Berube 2019 | 10.1093/pm/pnz00 | Montreal, Canada | LE trauma | 56 | 39% | 47±19, 38±14 |  | Leaflet/Pamphlet/Booklet | Print | | No |
| Bodes Pardo 2018 | [10.1016/j.apmr.2017.10.016](https://doi.org/10.1016/j.apmr.2017.10.016) | Madrid, Spain | cNSLBP | 56 | 79% | 49.2±10.5. 44.9±9.6 |  | Multiple (see description) | Combo | | No |
| Bossen 2013 | 10.2196/jmir.2662 | Netherlands | Hip or Kn OA | 199 | 65% | 62±5.7 |  |  | Digital | | No |
| Bostrøm 2023 | 10.2196/47284 | Oslo, Norway | Chronic pain | 259 | 81% | 49 (range 22-78) | App |  | Digital | | Yes (available online) |
| Braun 2022 | 10.3390/ijerph192113858 | Germany | Chronic pain | 89 | 64% | 56.98 ± 8.65 | Website/Blog |  | Digital | | No |
| Brison 2005 | [10.1097/01.brs.0000174115.58954.17](http://dx.doi.org.ucd.idm.oclc.org/10.1097/01.brs.0000174115.58954.17) | Kingston/Ottawa/Edmonton, Canada | WAD | 405 | 64% | 38.4 (16-85); 38.4 (17-81) | Video (or film) |  | Film | | No |
| Burton 1999 | <https://doi.org/10.1097/00007632-199912010-00010> | NE England, UK | NSLBP new episode, ± l e g pain | 162 | 55% | 42.6±10.9; 44.7±12.2 | Leaflet/Pamphlet/Booklet | Leaflet/Pamphlet/Booklet | Print | | No |
| Chenot 2019 | 10.1007/s00482-019-0362-6 | Gottingen & Berlin; Germany | Acute LBP | 364 | 57% | 40.3±11.3, 38.4±11.0 | Multiple (see description) | Leaflet/Pamphlet/Booklet | Print | | Yes (available online) |
| Cherkin 2001 | <https://doi.org/10.1001/archinte.161.8.1081> | Washington, USA | LBP | 262 | 58% | 44.9±11.5 |  | Multiple (see description) | Combo | | No |
| Chimenti 2023 | 10.1097/j.pain.0000000000002720 | USA | achilles tendinopathy | 66 | 56% | Mean 43.4 years, SD 15.5 | Multiple (see description) |  | Combo | | Yes (included in open access article) |
| Coudeyre 2006 | <https://doi.org/10.1016/j.annrmp.2006.05.003> | Montpellier, France | subacute or chronic LBP | 142 | 54% | 43.06±11.42; 44.98±10.4 | Leaflet/Pamphlet/Booklet |  | Print | | Yes (cited in article but requires purchase) |
| Coudeyre 2007 | 10.1371/journal.pone.0000706 | France | Acute LBP | 2337 | 43% | 45±12 | Book |  | Print | | Yes (cited in article but requires purchase) |
| Cramer 2013 | 10.1097/AJP.0b013e318251026c | Essen, Germany | NSNP Neck pain | 51 | 82% | 47.8±10.4 | Leaflet/Pamphlet/Booklet | Manual/Workbook | Print | | Yes (provided by authors on request) |
| Cuesta-Vargas 2011 | 10.1097/PHM.0B013E31821A71D0 | Malaga Spain | cNSLBP | 49 | 56% | 38.4 ± 11.3 | Leaflet/Pamphlet/Booklet | Leaflet/Pamphlet/Booklet | Print | | No |
| da Silva 2015 | 10.1682/JRRD.2014.08.0199 | Natal, Brazil | Knee OA | 30 | 87% | 57±6.01, 60±7.76 |  | Leaflet/Pamphlet/Booklet | Print | | No |
| Darnall 2020 | <https://doi.org/10.2196/17293> | USA | Multi L B P & Fibro | 74 | 30% |  | other (see description) |  | Digital | | No |
| Davis 2013 | <https://doi-org.ucd.idm.oclc.org/10.1007/s12160-013-9513-7> | Arizona, USA | Fibromyalgia | 79 | 99% | 46.14 (22–81) | Website/Blog |  | Digital | | No |
| de Boer 2014 | 10.1002/ejp.509 | Groningen, Netherlands | Chronic pain | 50 | 64% | 52.1±11.2; (19-75) | Manual/Workbook | Multiple (see description) | Combo | | No |
| Deegan 2023 | <https://dx.doi.org/10.1097/ajp.0000000000001126> | Ireland | Chronic pain | 96 | 82% |  | Multiple (see description) | Website/Blog | Digital | | No |
| Derebery 2009 | <https://doi.org/10.1097/brs.0b013e318193c9eb> | Texas/Oklahoma/New Mexico, USA | Neck pain | 552 | 43% | 37.4±11.5; 38.2±11.5 | Leaflet/Pamphlet/Booklet | Leaflet/Pamphlet/Booklet | Print | | No |
| Diab 2022 | PMID: 36288581 | NC, USA | Chronic pain | 60 | 82% | 52.1±17.8 | Website/Blog | Website/Blog | Digital | | Yes (available online) |
| DiGiovanni 2003 | 10.2106/00004623-200307000-00013 | Rochester, USA | Chronic heel pain LE | 101 | 67% | 46.5±7.5, (23-60) | Video (or film) | Video (or film) | Film | | No |
| Dobscha 2008 | 10.1111/j.1526-4637.2008.00457.x | Portland, OR, USA | Chronic msk pain or chronic pain | 401 | 8% | 61.7±11.8 | Manual/Workbook |  | Combo | | Yes (provided by authors on request) |
| Doering 2000 | 10.1097/00006842-200005000-00010 | Innsbruck, Austria | Hip OA (pre-op) | 100 | 38% | 58.7 ± 10.8; 60.4 ± 8.7 | Video (or film) |  | Film | | No |
| Dowd 2015 | <https://doi.org/10.1097/ajp.0000000000000201> | Galway, Ireland | Chronic pain | 124 | 90% | 44.53±12.25 (19-76) | Website/Blog |  | Digital | | No |
| Fioratti 2022 | 10.2196/35743 | Brazil | Chronic pain | 65 | 66% | 39.5 (SD 11.3) | Website/Blog | Website/Blog | Digital | | No |
| Frost 1995 | 10.1136/bmj.310.6973.151 | Headington, UK | cNSLBP | 71 | 52% | 34.2±9.4, 38.2±9.3 | Video (or film) | Video (or film) | Film | | No |
| Galan-Martin 2020 | 10.3390/jcm9041201 | Castilla & León, Spain | Chronic Spinal Pain | 170 | 80% |  | Book |  | Print | | No |
| Garcia 2022 | <https://doi.org/10.1016/j.jpain.2021.12.002> | USA | chronic LB±radicular pain (non- specific, radicular, associated with other spinal causes) | 179 | 77% | 51.5±13.5, 51.4±12.9 | Video (or film) | Video (or film) | Digital | | No |
| Garcia-Palacios 2015 | 10.1097/AJP.0000000000000196 | Castellon, Spain | Fibromyalgia | 61 | 100% | 50.48±9.78 (23-70) | Video (or film) |  | Digital | | No |
| Gardner 2019 | 10.1136/ bjsports-2018-100080 | Sydney, Australia | cLBP | 75 | 57% | 44±12.5, 45±13.8 | Manual/Workbook |  | Print | | Yes (included in institutional article) |
| Gasslander 2022 | 10.1080/16506073.2022.2065528 | Sweden |  | 187 | 73% | 45.9 years (19–64, SD = 11.2) | Website/Blog |  | Digital | | No |
| George 2009 | <https://doi.org/10.1007/s00586-009-1016-7> | Fort Sam Houston, Texas, USA | LBP | 3792 | 29% | 22±4.4 | Book |  | Print | | Yes (cited in article but requires purchase) |
| Gibbs 2022 | 10.1177/02692155221095484 | Sydney, Australia | Chronic LBP | 64 | 44% | 35.6±12.4; 33.5±11.9 | Video (or film) | Video (or film) | Digital | | Yes (available online) |
| Giro 2016 | <https://doi-org.ucd.idm.oclc.org/10.1016/j.prosdent.2016.03.021> | Sao Paulo, Brazil | Temperomandibular disorders TMJ | 42 | 100% | 36.4±8.8 | Video (or film) |  | Digital | | No |
| Grande-Alonso 2019 | 10.1093/pm/pnz093 | Spain | cNSLBP | 50 | 56% | 39.88±13.2; 38.29±13.1 |  | Powerpoint slides | Digital | | No |
| Groenveld 2023 | 10.1097/AJP.0000000000001110 | Netherlands | Chronic LBP | 41 | 83% | Mean 51.5 years, SD 11.9 | other (see description) |  | Digital | | No |
| Hauser-Ulrich 2020 | 10.2196/15806 | Germna-speaking Switzerland | ongoing pain / chronic pain | 102 | 80% | 43.7±12.7 | App |  | Digital | | No |
| Heapy 2017 | 10.1001/jamainternmed.2017.0223 | USA | chronic back pain | 125 | 22% | 57.9±11.6 | Manual/Workbook | Manual/Workbook | Combo | | No |
| Hochlehnert 2006 | 10.1016/j.pec.2005.02.014 | Germany | Fibromyalgia | 75 | 93% | 49.85±10.42 | Website/Blog | Website/Blog | Digital | | No |
| Hrkać 2022 | 10.1186/s12891-022-05908-3 | Mostar, Bosnia & Herzegovina | cNSLBP | 180 | 63% | 49.3 (SD 11.7) |  | Leaflet/Pamphlet/Booklet | Print | | Yes (included in open access article) |
| Ibrahim 2018 | <https://doi.org/10.12965%2Fjer.1836348.174> | Tsakuwa, Nigeria | CLBP | 30 | 20% | 48.5±14.9, 50.3±9.09, 49.9±8.82 | Multiple (see description) |  | Combo | | No |
| Ibrahim 2023 | 10.1186/s12891-022-06108-9 | Nigeria | chronic LBP | 120 | 40% | 46.0 [14.7] | Leaflet/Pamphlet/Booklet |  | Print | | Yes (included in open access article) |
| Janevic 2022 | <https://doi.org/10.1093/geront/gnac010> | Detroit, USA | Chronic Pain | 46 | 89% | 72.1±7.2; (60-90) | Multiple (see description) |  | Digital | | Yes (available online) |
| Jassi 2021 | 10.1016/j.apmr.2021.03.007 | Brazil | cLBP | 120 | 54% | 32.1±12.9, 28.4±11.4, 28.4±10.9 |  | Leaflet/Pamphlet/Booklet | Print | | No |
| Javdaneh 2021 | 10.3390/ ijerph18168848 | Tehran, Iran | NSNP Neck pain | 72 | 49% | 31.18±6.37, 33.45±7.08, 33.7±8.13 | Powerpoint slides | Leaflet/Pamphlet/Booklet | Combo | | No |
| Jay 2014 | 10.1155/2014/790937 | Copenhagen, Denmark | Multi: Neck or shoulder pain | 38 | 100% | 47±10; 43±6 | Video (or film) | Video (or film) | Combo | | No |
| Jinnouchi 2023 | 10.1093/mr/roac009 | Japan | chronic knee pain | 46 | 89% | Approximately 70 years old | Book | Book | Print | | Yes (included in institutional article) |
| Khosrokiani 2022 | <https://doi.org/10.1177/02692155211038099> | Tehran, Iran | Chronic neck pain | 113 | 67% | 39±5 |  | Leaflet/Pamphlet/Booklet | Print | | No |
| KIm 2021 | <https://doi.org/10.1097/jnr.0000000000000455> | Korea | LBP | 43 | 74% | 61.3±11.5; 54.5±12.8 | Leaflet/Pamphlet/Booklet | Leaflet/Pamphlet/Booklet | Print | | No |
| Kisaalita 2016 | 10.1016/j.jpain.2015.10.017 | Florida, USA | Chronic Msk pain / Chronic pain | 57 | 70% | 42.12±19.16 | Powerpoint slides | Powerpoint slides | Digital | | No |
| Ko 2013 | dx.doi.org/10.2106/JBJS.L.00964 | Sydney, Australia | Knee OA receiving TKA | 249 | #VALUE! |  |  | Multiple (see description) | Combo | | No |
| Kohns 2020 | 10.1097/AJP.0000000000000857 | Michigan, USA | Chronic Msk pain / Chronic pain | 104 | 73% | 44.35±14.71 | Video (or film) |  | Digital | | Yes (available online) |
| Kuvačić 2018 | <https://doi.org/10.1016/j.ctcp.2018.03.008> | ?, Croatia | cLBP | 30 | 47% | 34.2 (25-42) |  | Leaflet/Pamphlet/Booklet | Print | | No |
| Kwok 2016 | <http://dx.doi.org/10.1080/07317115.2016.1171818> | Hong Kong | chronic knee pain | 46 | #VALUE! | not reported |  | Leaflet/Pamphlet/Booklet | Print | | No |
| Lamb 2010 | 10.1016/S0140- 6736(09)62164-4 | England, UK | subacute or cLBP | 701 | 60% | 54 (18-85) | Multiple (see description) |  | Print | | Yes (cited in article but requires purchase) |
| LeFort 1998 | <https://doi.org/10.1016/S0304-3959(97)00190-5> | St John's, Canada | chronic pain | 110 | 75% | 39(24-57),4026-60) | Multiple (see description) |  | Combo | | No |
| Li 2020 | 10.3390/ijerph17144966 | Hong Kong | chronic pain | 64 | 73% | 71.7±14.6 (61-92) | Multiple (see description) | Leaflet/Pamphlet/Booklet | Combo | | No |
| Li 2020 | <https://doi.org/10.2196/15071> | China | General Pain | 95 | 72% | (16-60) |  | Leaflet/Pamphlet/Booklet | Print | | No |
| Lin 2018 | <https://doi.org/10.2196/12015> | Germany | chronic pain | 115 | 71% | 50.42±13.67 (18-76) | Video (or film) |  | Digital | | No |
| Linton 2000 | 0.1097/00007632-200011010-00017 | Sweden | cLBP | 243 | 71% | 44 |  | Multiple (see description) | Print | | No |
| Little 2001 | 10.1097/00007632-200110010-00003 | deprived urban area, Southampton, UK | new epsiode LBP | 311 | not reported | 47±14, 42±14, 47±14, 47±12 | Leaflet/Pamphlet/Booklet | Leaflet/Pamphlet/Booklet | Print | | No |
| Lorig 2008 | <https://doi.org/10.1002/art.23817> | USA | Multi Osteo Arthritis or fibro | 866 | 90% | 52.5±12.2; 52.2±10.9 (22-89) | Multiple (see description) |  | Combo | | No |
| Louw 2014 | 10.1097/BRS.0000000000000444 | USA | lumbar radiculopathy, undergoing surgery | 67 | not reported | 49.59, 49.65 | Leaflet/Pamphlet/Booklet | Leaflet/Pamphlet/Booklet | Print | | No |
| Malfliet 2018 | 10.1001/jamaneurol.2018.0492 | Ghent & Brussels, Belgium | chronic non-specific spinal pain | 120 | 61% | 39.91±11.95, 40.53±12.88 | Leaflet/Pamphlet/Booklet | Leaflet/Pamphlet/Booklet | Combo | | No |
| Marcus 2007 | <https://doi.org/10.1001/archinte.167.9.944> | Providence & Pittsburgh, USA | Sedentary | 249 | 83% |  | Website/Blog | Manual/Workbook | Combo | | No |
| Mecklenburg 2018 | <http://dx.doi.org/10.2196/jmir.9667> | USA | chronic knee pain | 155 | 37% | 46±12 | App | Website/Blog | Digital | | No |
| Meeus 2010 | 10.1016/j.apmr.2010.04.020 | Belgium | Chronic fatigu e syndrome, chronic widespread pain/ chronic pain | 48 | 83% | 38.3±10.6, 42.3±10.2 | Multiple (see description) |  | Print | | Yes (provided by authors on request) |
| Mellor 2018 | <https://doi.org/10.1136/bmj.k1662> | Brisbane & Melbourne, Australia | Gluteal tendinopathy | 204 | 82% | 54.8±8.8 | Multiple (see description) |  | Combo | | No |
| Michaleff 2014 | <http://dx.doi.org/10.1016/S0140-6736(14)60457-8> | Sydney & Brisbane, Australia | chronic WAD Gr 1 or 2 | 170 | 64% | 42.6±12.3, 43.1±12.7 | Leaflet/Pamphlet/Booklet | Leaflet/Pamphlet/Booklet | Print | | No |
| Michelotti 2012 | <https://doi.org/10.14219/jada.archive.2012.0018> | Naples, Italy | TMJ pain | 44 | 77% | 31.2±11.8 (18-53) | Leaflet/Pamphlet/Booklet | Leaflet/Pamphlet/Booklet | Print | | No |
| Michou 2022 | 10.46497/ArchRheumatol.2022.8965 | Quebec, Canada | Rheumatoid Arthritis | 107 | 75% | 60.2±10.4 (54-71) | Video (or film) |  | Digital | | No |
| Miyamoto 2018 | http://dx.doi.org/10.1136/ bjsports-2017-098825 | Sao Paulo, Brazil | NSCLBP | 296 | 76% | 47-48.9± 11.5-16.6 |  | Leaflet/Pamphlet/Booklet | Print | | No |
| Morcillo-Muñoz 2022 | <http://dx.doi.org/10.2196/36114> | Spain | Chronic Pain | 194 | 80% | 51.2±11.2; 50.3±10.2 | App | App | Digital | | No |
| Moseley 2002 | <https://doi-org.ucd.idm.oclc.org/10.1016/S0004-9514(14)60169-0> | Brisbane, Australia | LBP | 57 | 59% | 43±7; 38±7 | Manual/Workbook |  | Print | | No |
| Mukhtar 2022 | 10.1080/09638288.2021.1988155 | Nigeria | chronic neck pain | 53 | 49% | Mean 36.4 years, SD 14.16 | Multiple (see description) |  | Combo | | Yes (included in open access article) |
| Nambi 2020 | <https://doi.org/10.1155/2020/2981273> | Al-Kharj, Saudi Arabia | LBP | 45 | 0% | 20.23±1.6; 21.25±1.2; 20.78±1.6 | other (see description) |  | Digital | | No |
| Nambi 2021 | 10.3233/THC-202301 | Al-Kharj, Saudi Arabia | cLBP | 60 | 0% | 23.2±1.5, 22.8±1.6, 23.3±1.5 | Video (or film) |  | Digital | | No |
| Nicholas 2017 | <http://dx.doi.org/10.1097/j.pain.0000000000000729> | Sydney, Australia | chronic pain conditions | 141 | 63% | 73.9±6.5 (65-87) | Manual/Workbook | Manual/Workbook | Print | | No |
| Nordin 2016 | <http://dx.doi.org/10.2196/jmir.5634> | Norrbotten county, Sweden | persistent Msk pain / chronic pain | 99 | 85% | 44±10, 42±11 | Website/Blog |  | Digital | | No |
| O'Connor 2016 | <http://dx.doi.org/10.2196/ijmr.4295> | Florida, USA | undergoing THR or TKR / OA | 53 | 58% | 67.4±10.3, 63.1±10.7 | Video (or film) | Leaflet/Pamphlet/Booklet | Combo | | No |
| O’Keeffe 2020 | http://dx.doi.org/10.1136/ bjsports-2019-100780 | Ireland | cLBP | 206 | 74% | 47±13.2, 50.6±14.9 | Leaflet/Pamphlet/Booklet |  | Combo | | Yes (provided by authors on request) |
| Oliveira 2006 | 10.1097/01.brs.0000224172.45828.e3 | California, USA | cervical strain / whiplash / WAD | 126 | 58% | 39.38, 35.86 | Video (or film) |  | Combo | | No |
| Pacella-LaBarbara 2020 | 10.1111/acem.14000 | USA | WAD | 64 | 63% | Mean 37.0 years, SD 12.71 | App |  | Digital | | Yes (available online) |
| Pach 2022 | <http://dx.doi.org/10.2196/31482> | Berlin, Germany | chronic neck pain | 220 | 70% | 38.9±11.3 | App | App | Digital | | No |
| Peters 2017 | 10.1097/AJP.0000000000000494 | Multi: BEL+NED | Chronic pain | 276 | 85% | 48.6±12 | Multiple (see description) |  | Combo | | No |
| Rabiei 2020 | 10.1111/papr.12963 | Iran | cLBP | 80 | 49% | 42.46±9.7, 44.19±8.79 | Multiple (see description) |  | Print | | No |
| Rafiq 2021 | <https://doi.org/10.1155/2021/6672274> | Lahore, Pakistan | Knee OA + obesity | 50 | 54% | 53.12±5.41 | Leaflet/Pamphlet/Booklet | Leaflet/Pamphlet/Booklet | Print | | No |
| Rini 2015 | <https://doi.org/10.1097%2Fj.pain.0000000000000121> | Johnston County, NC, USA | Hip or Kn OA | 113 | 81% | 67.62±9.45 (38-90) | App |  | Digital | | No |
| Riva 2014 | <http://dx.doi.org/10.2196/jmir.3474> | Canton Ticino, Switzerland | chronic back pain | 51 | 51% | 51±14.1, 44±13.6 | Website/Blog | Website/Blog | Digital | | No |
| Rodriguez Sánchez-Laulhé 2022 | <http://dx.doi.org/10.2196/35462> | Seville, Spain | Rheumatoid Arthritis hand pain | 36 | 61% | (43-78) | App | Leaflet/Pamphlet/Booklet | Combo | | No |
| Rodrıguez-Torres 2020 | <https://doi.org/10.1016/j.apmr.2020.02.019> | Granada, Spain | chronic pelvic pain | 38 | 100% | 42.93±8.22, 43.50±7.86 |  | Leaflet/Pamphlet/Booklet | Print | | No |
| Roseen 2023 | 10.1007/s11606-023-08037-2 | USA | Chronic LBP | 120 | 9% | Mean 55.5 years, SD 16.9 | Video (or film) | Book | Combo | | Yes (included in open access article) |
| Ruehlman 2012 | 10.1016/j.pain.2011.10.025 | New England, USA | Chronic pain | 305 | 64% | 44.93 (19-78) | Website/Blog |  | Digital | | No |
| Ryan 2010 | 10.1016/j.math.2010.03.003 | Glasgow, UK | cLBP | 38 | 66% | 45.2±11.9; 45.5±9.5 | Multiple (see description) | Multiple (see description) | Print | | No |
| Sandal 2021 | 10.1001/jamainternmed.2021.4097 | Multi (DEN + NOR)) | LBP | 461 | 55% | Mean 47.5 years, SD 14.7, Range 18–86 | App |  | Digital | | No |
| Sander 2020 | 10.1001/jamapsychiatry.2020.1021 | Germany | Back pain | 295 | 62% | Mean 52.8 years, SD 7.7 | Website/Blog |  | Digital | | No |
| Sandhu 2023 | 10.1001/jama.2023.6454 | England, UK | Chronic pain | 608 | 60% | 61 [12.9] | Multiple (see description) | Multiple (see description) | Combo | | Yes (available online) |
| Saper 2017 | 10.7326/M16-2579 | Boston, USA | cLBP | 320 | 64% | 46±10.7 | Multiple (see description) |  | Combo | | Yes (cited in article but requires purchase) |
| Saw 2016 | 10.1186/s12891-016-1088-6 | Western Cape & Gauteng, South Africa | hip & kn OA | 74 | 81% | 60.72±5.54 | Manual/Workbook |  | Print | | No |
| Schaller 2016 | 10.1177/0269215515618730 | Germany | cLBP | 412 | 31% |  | Website/Blog | Website/Blog | Digital | | No |
| Schmidt 2021 | <https://doi.org/10.1186/s12891-021-04236-2> | Freiburg & Bad Oldesloe, Germany | NSCLBP | 110 | 69% | 52.4±12.81, 52.5±13.63 |  | Leaflet/Pamphlet/Booklet | Print | | No |
| Serrat 2021 | <https://doi.org/10.3390/ijerph181910300> | Barcelona, Spain | Fibromyalgia | 151 | 93% | 54.35±8.68 (22-76) | Video (or film) |  | Digital | | No |
| Serrat 2022 | https://doi.org/10.1016/j.brat.2022.104188 | Barcelona, Spain | Fibromyalgia | 330 | 62% | 53.48±8.93, 52.78±8.64,  52.54±9.78 | Video (or film) |  | Digital | | No |
| Sharpe 2023 | 10.1097/j.pain.0000000000002784 | Sydney, Australia | Chronic pain | 288 | 85% | 49.34 (14.96) 49.93 (13.92) 48.68 (13.30) 49.52 (13.38) | Video (or film) |  | Digital | | No |
| Shaygan 2022 | <https://doi.org/10.1371/journal.pone.0269785> | Shiraz, Iran | NSLBP | 90 | 77% | 50.52±10.3 | other (see description) |  | Digital | | No |
| Shaygan 2022 | 10.17533/udea.iee.v40n1e13 | Shiraz, Iran | chronic NSLBP | 60 | 75% | unclear reporting | Multiple (see description) |  | Digital | | No |
| Sherman 2005 | <https://doi.org/10.7326/0003-4819-143-12-200512200-00003> | Washington & Idaho, USA | LBP | 101 | 65% | 44±13 | Leaflet/Pamphlet/Booklet | Book | Print | | Yes (cited in article but requires purchase) |
| Sherman 2011 | 10.1001/archinternmed.2011.524 | Puget Sound, WA, USA | Back pain | 228 | 64% | 48.4±9.8 | Video (or film) | Book | Combo | | Yes (cited in article but requires purchase) |
| Shpaner 2014 | <http://dx.doi.org/10.1016/j.nicl.2014.07.008> | USA | Chronic pain | 38 | 76% | 43.6±13.7,  39.2±14.1 |  | Leaflet/Pamphlet/Booklet | Print | | No |
| Simister 2018 | <https://doi-org.ucd.idm.oclc.org/10.1016/j.jpain.2018.02.004> | Manitoba, Canada | Fibromyalgia | 67 | 95% | 39.7 ± 9.36 (18-64) | Website/Blog | Video (or film) | Digital | | No |
| Simula 2021 | <https://doi.org/10.1186/s12875-021-01529-2> | Finland | LBP | 415 | 64% | 41.4±12.8; 44.6±12.6 | Leaflet/Pamphlet/Booklet |  | Print | | Yes (available online) |
| Singh 2018 | <https://doi.org/10.1097/BCO.0000000000000632> | London, Canada | undergoing elective foot/ank surgery LE | 80 | 79% | 50.68 (20-65) | Leaflet/Pamphlet/Booklet |  | Print | | Yes (available online) |
| Skillgate 2020 | <https://doi.org/10.1016/j.msksp.2019.102070> | Stockholm, Sweden | subacute to chronic NSNP / neck pain | 619 | 69% | 46 |  | Leaflet/Pamphlet/Booklet | Print | | No |
| Skou 2015 | <http://dx.doi.org/10.1016/j.joca.2015.04.021> | northern Denmark | Knee OA | 100 | 51% | 64.8±8.7,  67.1±9.1 | Powerpoint slides |  | Combo | | Yes (provided by authors on request) |
| Skou 2015 | 10.1056/NEJMoa1505467 | Frederikshavn & Farsø, Denmark | Knee OA | 100 | 62% | 65.8±8.7, 67±8.7 | Video (or film) | Video (or film) | Digital | | No |
| Sorensen 2010 | <https://doi.org/10.1186/1471-2474-11-212> | Denmark | cLBP | 210 | 52% | median 40 (IQR 33-47); 40(3-48); 38(32-47) | Powerpoint slides |  | Digital | | No |
| Syed 2018 | <https://doi.org/10.1016/j.jse.2018.02.039> | USA | undergoing arthroscopic rotator cuff repair UE | 134 | 32% | 58±9.4; 59.2±9.2 | Video (or film) |  | Digital | | Yes (included in institutional article) |
| Tejera 2020 | <https://doi.org/10.3390/ijerph17165950> | Spain | NSCNP Neck pain | 44 | 52% | 29.7±10.81 | other (see description) |  | Digital | | No |
| Thompson 2016 | <https://doi-org.ucd.idm.oclc.org/10.1016/j.physio.2015.04.008> | Manchester, UK | CNSNP chronic neck pain | 200 | 46% | 45.8±12.6; 49.2±14.5 |  | Leaflet/Pamphlet/Booklet | Print | | Yes (provided by authors on request) |
| Thorn 2018 | 10.7326/M17-0972 | western Alabama, USA | chronic pain | 290 | 71% | 50.6±8.9; (19-71) | Multiple (see description) |  | Combo | | Yes (available online) |
| Timmerman 2016 | 10.1093/pm/pnw013 | Netherlands | chronic pain | 92 | 55% | 60.2±14; 58.5±12.7 | Multiple (see description) |  | Combo | | No |
| Traeger 2019 | 10.1001/jamaneurol.2018.3376 | Australia | acute LB±radicular pain | 202 | 51% | 46.5±14.7; 43.8±14.1 | Manual/Workbook |  | Print | | Yes (included in institutional article) |
| Triano 1995 | 10.1097/00007632-199504150-00013 | Texas, USA | recurring or cLBP | 170 | 45% | 42±14.4 |  | Leaflet/Pamphlet/Booklet | Print | | No |
| Tse 2014 | http://dx.doi.org/10.1016/ j.pmn.2013.08.003 | Hong Kong | Msk pain / chronic pain | 396 | 80% | 85.44±6.29 | Leaflet/Pamphlet/Booklet |  | Print | | No |
| Tse 2023 | 10.1002/nop2.1915 | Hong Kong | Chronic pain | 71 | 72% | 72.10± 8.71,  75.17±10.46 | Multiple (see description) | Leaflet/Pamphlet/Booklet | Combo | | No |
| Ünal 2020 | <https://doi.org/10.1016/j.jbmt.2020.07.014> | Turkey | cLBP | 40 | 50% | 41.25±9.12; 42.6±7.96 |  | Leaflet/Pamphlet/Booklet | Print | | No |
| Valenza 2017 | 10.1177/0269215516651978 | Granada, Spain | CNSLBP | 54 | 77% | 37.62±12.14; 40.27±15.84 |  | Leaflet/Pamphlet/Booklet | Print | | No |
| Valiente-Castrillo 2021 | 10.1177/0964528420920300 | Spain | chronic neck pain | 60 | 85% | 40.8±8.06; 40.2±11.37; 42.1±9.07 | Video (or film) |  | Digital | | Yes (available online) |
| van Ittersum 2013 | 10.1111/papr.12137 | Belgium | Fibromyalgia | 105 | 93% | 46.5±9.3 | Leaflet/Pamphlet/Booklet | Leaflet/Pamphlet/Booklet | Print | | No |
| van Oosterwijck 2013 | 10.1097/AJP.0b013e31827c7a7d | Belgium | Fibromyalgia | 30 | 87% | 48.5±9.5; 45.9±11.5 | Multiple (see description) | Multiple (see description) | Combo | | No |
| Vanti 2019 | 10.1093/ptj/pzz056 | Bologna, Italy | chronic NSNP / neck pain | 70 | 84% | 48.3±9.9; 50.9±7.8 | Manual/Workbook | Leaflet/Pamphlet/Booklet | Print | | Yes (included in institutional article) |
| Vicente-Mampel 2022 | 10.3390/ijerph191911855 | Spain | chronic pain MSK | 50 | 28% | PET: 75.79 ± 5.92 years, CG: 74.07 ± 6.27 years | Multiple (see description) |  | Combo | | No |
| Walsh 2020 | 10.1016/j.msksp.2020.102271 | SW England, UK | arthritic Knee, Hip, Low Back / OA | 349 | 62% | 66.5±8.4; 66.3±8.1 | Multiple (see description) |  | Print | | Yes (provided by authors on request) |
| Wegwarth 2022 | <https://doi.org/10.1016/j.pec.2021.10.002> | Berlin, Germany | Chronic pain | 300 | 49% | gives n per category | Website/Blog | Website/Blog | Digital | | No |
| Westenberg 2018 | 10.1007/s11999.0000000000000086 | ?, USA | pain in an upper limb ortho population / UE | 125 | 50% | 55±15 | Website/Blog | Leaflet/Pamphlet/Booklet | Combo | | Yes (available online) |
| Wiklund 2022 | <http://dx.doi.org/10.2196/29258> | Sweden | Chronic Pain with insomnia | 54 | 83% | 49.3±12.3 | Multiple (see description) | Multiple (see description) | Digital | | No |
| Williams 2010 | <https://doi.org/10.1016%2Fj.pain.2010.08.034> | Sioux Falls, South Dakota, USA | Fibromyalgia | 118 | 95% | 50±11.5 | Website/Blog |  | Digital | | No |
| Williams 2022 | <http://dx.doi.org/10.1097/j.pain.0000000000002586> | Washington, USA | Chronic pain | 328 | 26% | 53.5 (13.5), 51.0 (12.6), 55.0 (13.0) | Multiple (see description) |  | Combo | | No |
| Wilson 2023 | 10.1097/j.pain.0000000000002785 | USA | Chronic pain | 402 | 69% | Mean 56.7 years, SD 11.0 | Website/Blog |  | Digital | | No |
| Yeh 2022 | <https://doi.org/10.3390/ijerph192214875> | TX, USA | Chronic pain | 37 | 68% | 58.76 (12.04) (22-68) 53.38 (14.79) (36-80) | App |  | Digital | | No |
| Yuan 2021 | <https://doi.org/10.1016/j.bjpt.2020.10.003> | Sao Paulo, Brazil | Fibromyalgia | 40 | 98% | 43±10.1 | App | Book | Combo | | Yes (provided by authors on request) |
| Zheng 2022 | <https://doi.org/10.1186/s13063-022-06116-z> | China | Chronic LBP | 40 | 65% | 34.0±14.4; 34.9±14.5 | Video (or film) | Video (or film) | Digital | | No |
| Ziadni 2021 | 10.2196/29672 | USA | chronic pain | 101 | 69% | 48.6±14.1; 50.9±13.7; (26-78) | Multiple (see description) |  | Combo | | No |

**APPENDIX E - RoB-2 Tool**

| **Study** | **Risk of bias arising from the randomization process** | **Risk of bias due to deviations from the intended interventions (effect of assignment to intervention)** | **Missing outcome data** | **Risk of bias in measurement of the outcome** | **Risk of bias in selection of the reported result** | **Overall risk of bias** |
| --- | --- | --- | --- | --- | --- | --- |
| Aguirrezabal 2019 | Some concerns | Some concerns | High | Low | Some concerns | High |
| Alasfour 2022 | Low | Low | Some concerns | Some concerns | Low | Some concerns |
| Albaladejo 2010 | Low | Low | Low | Low | Low | Low |
| Almhdawi 2020 | High | Low | Some concerns | Low | Some concerns | High |
| Amaral 2020 | Low | Low | Low | Some concerns | Some concerns | Some concerns |
| Amer-Cuenca 2020 | Low | High | High | Low | Low | High |
| Archer 2016 | Low | Low | Low | Low | Low | Low |
| Areeudomwong 2017 | Low | Low | Low | Low | Some concerns | Some concerns |
| Ariza-Mateos 2020 | Some concerns | Low | Low | Low | Low | Some concerns |
| Bandak 2021 | Low | Low | Low | Low | Low | Low |
| Barrenengoa-Cuadra 2021 | Low | Low | Low | Some concerns | Low | Some concerns |
| Baumeister 2015 | Low | Low | Low | High | High | High |
| Baumeister 2021 | Low | Some concerns | Low | Low | Low | Low |
| Beltran-Alacreu 2015 | Low | Low | Low | Low | Low | Low |
| Bennell 2017 | Some concerns | Low | Low | Some concerns | Low | Some concerns |
| Berberoglu 2023 | Low | Low | Low | Low | Some concerns | Low |
| Berube 2019 | Low | Low | Low | Some concerns | Low | Some concerns |
| BodesPardo 2018 | Low | Low | Low | Some concerns | Low | Some concerns |
| Bossen 2013 | Low | Low | Some concerns | Low | Low | Some concerns |
| Bostrom 2023 | Low | Some concerns | Low | Low | Low | Low |
| Braun 2022 | Low | Some concerns | Low | Low | Low | Low |
| Brison 2005 | Low | Some concerns | Low | Low | Some concerns | Some concerns |
| Burton 1999 | Low | Low | Low | Low | Low | Low |
| Chenot 2019 | Low | Some concerns | Some concerns | Some concerns | Low | Some concerns |
| Cherkin 2001 | Low | Low | Low | Low | Low | Low |
| Chimenti 2023 | Some concerns | Low | Low | Some concerns | Low | Some concerns |
| Coudeyre 2006 | Low | Some concerns | High | Low | Some concerns | High |
| Coudeyre 2007 | Some concerns | Low | Low | Low | Low | Some concerns |
| Cramer 2013 | Some concerns | Low | Low | Low | Low | Some concerns |
| Cuesta-Vargas 2011 | Low | Low | Some concerns | Low | Low | Some concerns |
| Darnall 2020 | Low | Low | Low | Some concerns | Low | Some concerns |
| daSilva 2015 | Low | Low | Some concerns | Low | Low | Some concerns |
| Davis 2013 | Low | Low | Low | Some concerns | Low | Some concerns |
| deBoer 2014 | Low | Some concerns | Low | Some concerns | Some concerns | Some concerns |
| Deegan 2023 | Low | Some concerns | Low | Low | Low | Low |
| Derebery 2009 | Low | Some concerns | High | Some concerns | Some concerns | High |
| Diab 2022 | Low | Some concerns | Low | Low | Low | Low |
| DiGiovanni 2003 | Some concerns | Some concerns | High | Some concerns | Low | High |
| Dobscha 2008 | Low | Some concerns | Some concerns | Low | Some concerns | Some concerns |
| Doering 2000 | Some concerns | Low | Some concerns | Low | Low | Some concerns |
| Dowd 2015 | Low | Some concerns | Low | Some concerns | Some concerns | Some concerns |
| Fioratti 2022 | Low | Low | Low | Some concerns | Low | Low |
| Frost 1995 | Low | Low | Low | Low | Low | Low |
| Galan-Martin 2020 | Low | Low | Some concerns | Low | Low | Some concerns |
| Garcia 2022 | Low | Low | Low | Low | Low | Low |
| Garcia-Palacios 2015 | Low | High | Some concerns | Some concerns | Low | Some concerns |
| Gardner 2019 | Low | Low | Low | Low | Low | Low |
| Gasslander 2022 | Low | Some concerns | Low | Some concerns | Low | Some concerns |
| George 2009 | Some concerns | Low | Low | Some concerns | Low | Some concerns |
| Gibbs 2022 | Low | Low | Low | Some concerns | Some concerns | Some concerns |
| Giro 2016 | Low | Low | High | Low | Low | High |
| Grande-Alonso 2019 | Low | Low | Low | Low | Low | Some concerns |
| Groenveld 2023 | Low | Some concerns | Some concerns | Low | Low | Some concerns |
| Hauser-Ulrich 2020 | Low | Low | Some concerns | Some concerns | Low | Some concerns |
| Heapy 2017 | Low | Low | Some concerns | Some concerns | Low | Some concerns |
| Hochlehnert 2006 | Low | Low | Low | Some concerns | Low | Some concerns |
| Hrkać 2022 | Some concerns | Some concerns | Low | Some concerns | Low | Some concerns |
| Ibrahim 2018 | Low | Low | Some concerns | Low | Low | Some concerns |
| Ibrahim 2023 | Low | Some concerns | Low | Some concerns | Some concerns | Some concerns |
| Janevic 2022 | Some concerns | Low | Some concerns | High | Low | High |
| Jassi 2021 | Low | Some concerns | Some concerns | Low | Low | Some concerns |
| Javdaneh 2021 | Low | Low | Some concerns | Low | Some concerns | Some concerns |
| Jay 2014 | Low | Low | Low | Low | Low | Low |
| Jinnouchi 2023 | Low | Some concerns | Low | Low | Low | Some concerns |
| Khosrokiani 2022 | Low | Low | Low | Low | Low | Low |
| Kim 2021 | Some concerns | High | High | Low | Low | High |
| Kisaalita 2014 | Some concerns | Low | Low | Low | Some concerns | Some concerns |
| Ko 2013 |  |  |  |  |  |  |
| Kohns 2020 | Low | Some concerns | Low | Low | Some concerns | Some concerns |
| Kuvaƒçiƒá 2018 | Some concerns | Low | High | Some concerns | Low | High |
| Kwok 2016 | Some concerns | Low | Low | Some concerns | Some concerns | Some concerns |
| Lamb 2010 | Low | Low | Low | Low | Low | Low |
| Lefort 1998 | Low | Low | Low | Low | Low | Low |
| Li Y. 2020 | Low | Low | Low | Some concerns | Some concerns | Some concerns |
| Li Z. 2020 | Low | High | High | Some concerns | Some concerns | High |
| Lin 2018 | Low | High | Low | Some concerns | Low | High |
| Linton 2000 | Low | Some concerns | Some concerns | Some concerns | Some concerns | High |
| Little 2001 | Some concerns | Low | Some concerns | Some concerns | Some concerns | Some concerns |
| Lorig 2008 | High | Some concerns | Some concerns | Some concerns | Some concerns | High |
| Louw 2014 | Low | Low | Some concerns | Some concerns | Some concerns | Some concerns |
| Malfliet 2018 | Low | Low | Low | Low | Low | Some concerns |
| Marcus 2007 | Low | Some concerns | High | Some concerns | Some concerns | High |
| Mecklenburg 2018 | Some concerns | Low | Some concerns | Some concerns | Low | Some concerns |
| Meeus 2010 | Some concerns | Low | Low | Low | Some concerns | Some concerns |
| Mellor 2018 | Low | Low | Low | Low | Low | Low |
| Michaleff 2014 | Some concerns | Low | Low | Low | Low | Some concerns |
| Michou 2022 | Some concerns | Some concerns | Some concerns | Some concerns | Some concerns | High |
| Miyamoto 2018 | Low | Low | Low | Low | Low | Low |
| Morcillo-Mu√±oz 2022 | Low | Some concerns | High | Some concerns | High | High |
| Moseley 2002 | Low | Low | Some concerns | Low | Some concerns | Some concerns |
| Mukhtar 2022 | Low | Low | Some concerns | Low | Low | Low |
| Nambi 2020 | Some concerns | Low | Low | Some concerns | Some concerns | Some concerns |
| Nambi 2021 | Some concerns | Low | Low | Low | Some concerns | Some concerns |
| Nicholas 2017 | Low | Low | Some concerns | Low | Low | Some concerns |
| Nordin 2016 | Some concerns | Some concerns | Some concerns | Some concerns | Some concerns | High |
| O'Connor 2016 | High | Some concerns | High | Some concerns | High | High |
| O'Keeffe 2020 | Low | Some concerns | Low | Some concerns | Low | Some concerns |
| Oliveira 2006 | High | Some concerns | High | Some concerns | Some concerns | High |
| Pacella-LaBarba 2020 | Low | Low | Low | Some concerns | Low | Low |
| Pach 2022 | Some concerns | Some concerns | Some concerns | Some concerns | Low | Some concerns |
| Peters 2017 | High | Some concerns | Some concerns | Some concerns | Some concerns | High |
| Rabiei 2021 | Low | Some concerns | Some concerns | Some concerns | Some concerns | Some concerns |
| Rafiq 2021 | Some concerns | Low | Some concerns | Low | Low | Some concerns |
| Rini 2015 | Low | Low | Low | Some concerns | Some concerns | Some concerns |
| Riva 2014 | Some concerns | Low | Low | Low | Some concerns | Some concerns |
| Rodr√≠guez-Torres 2020 | Low | Low | High | Some concerns | Some concerns | High |
| Rodríguez Sánchez-Laulhé 2022 | Low | Some concerns | Some concerns | Some concerns | Low | Some concerns |
| Roseen 2023 | Low | Low | Some concerns | Some concerns | Low | Some concerns |
| Ruehlman 2012 | Some concerns | Some concerns | Some concerns | Some concerns | Some concerns | High |
| Ryan 2010 | Some concerns | Some concerns | Some concerns | Some concerns | Some concerns | Some concerns |
| Sandal 2021 | Low | Some concerns | Low | Some concerns | Low | Some concerns |
| Sander 2020 | Low | Low | Low | Low | Low | Low |
| Sandhu 2023 | Low | Some concerns | Low | Low | Low | Some concerns |
| Saper 2017 | Some concerns | High | Some concerns | Low | Low | High |
| Saw 2016 | High | Some concerns | Some concerns | Some concerns | Some concerns | High |
| Schaller 2016 | High | Some concerns | Some concerns | Some concerns | Low | High |
| Schmidt 2021 | Low | Some concerns | Some concerns | Some concerns | Some concerns | High |
| Serrat 2021 | Low | Some concerns | High | Some concerns | Some concerns | High |
| Serrat 2022 | Low | Some concerns | Low | Low | Low | Low |
| Sharpe 2023 | Low | Low | Low | Low | Low | Low |
| Shaygan 2022 | Some concerns | High | High | Some concerns | High | High |
| Shaygan 2022 | Low | Some concerns | Low | Some concerns | Low | Some concerns |
| Sherman 2005 | Low | Low | Some concerns | Low | Some concerns | Some concerns |
| Sherman 2011 | Low | Low | Low | Some concerns | Low |  |
| Shpaner 2014 | Some concerns | Some concerns | Some concerns | Some concerns | Some concerns | High |
| Simister 2018 | Low | Low | Some concerns | Some concerns | Low | Some concerns |
| Simula 2021 | Some concerns | Some concerns | High | Some concerns | Some concerns | High |
| Singh 2018 | Some concerns | Low | Some concerns | Some concerns | Some concerns | High |
| Skillgate 2020 | Low | Low | Some concerns | Some concerns | Low | Some concerns |
| Skou 2015 | Low | Low | Low | Low | Low | Low |
| Skou 2015b | Low | Low | Low | Low | Low | Low |
| Sorensen 2010 | Low | Some concerns | Low | Low | Low | Some concerns |
| Syed 2018 | Some concerns | Low | Some concerns | Some concerns | Some concerns | Some concerns |
| Tejera 2020 | Low | Some concerns | Some concerns | Low | Some concerns | Some concerns |
| Thompson 2016 | Low | Low | Some concerns | Some concerns | Some concerns | Some concerns |
| Thorn 2018 | Low | Low | Low | Low | Low | Low |
| Timmerman 2016 | Some concerns | Some concerns | Low | Some concerns | Some concerns | Some concerns |
| Traeger 2019 | Low | Low | Low | Low | Low | Low |
| Triano 1995 | Low | Some concerns | Some concerns | Some concerns | Some concerns | Some concerns |
| Tse 2014 | High | High | High | Some concerns | Some concerns | High |
| Tse 2023 | Low | Low | Low | Low | Low | Low |
| Unal 2020 | Some concerns | Some concerns | Low | Some concerns | Some concerns | Some concerns |
| Valenza 2017 | Low | Some concerns | Low | Some concerns | Some concerns | Some concerns |
| Valiente-Castrillo 2021 | Some concerns | Some concerns | High | Some concerns | High | High |
| vanIttersum 2014 | Low | Low | Low | Low | Some concerns | Some concerns |
| VanOosterwijck 2013 | Low | Low | Low | Low | Some concerns | Some concerns |
| Vanti 2019 | Low | Some concerns | Low | Some concerns | High | High |
| Vicente-Mampel 2022 | Some concerns | Some concerns | Low | Low | Some concerns | Some concerns |
| Walsh 2020 | Low | Low | Low | Low | Low | Low |
| Wegwarth 2022 | Low | Low | Some concerns | Low | Low |  |
| Westenberg 2018 | Some concerns | Low | Low | Some concerns | High | High |
| Wiklund 2022 | Some concerns | Low | Low | Some concerns | Some concerns | Some concerns |
| Williams 2010 | Low | Low | Low | Some concerns | Some concerns | Some concerns |
| Williams 2022 | Low | Some concerns | Low | Low | Some concerns | Some concerns |
| Yeh 2022 | Low | Some concerns | Low | Low | Low | Low |
| Yuan 2021 | Low | Low | Some concerns | Some concerns | Low | Some concerns |
| Zheng 2022 | High | Some concerns | Low | Low | Some concerns | High |
| Ziadni 2021 | Some concerns | Low | Low | Some concerns | Low | Some concerns |

| Study | Risk of bias arising from the randomization process | Risk of bias due to deviations from the intended interventions (effect of assignment to intervention) | Missing outcome data | Risk of bias in measurement of the outcome | Risk of bias in selection of the reported result | Overall risk of bias |
| --- | --- | --- | --- | --- | --- | --- |
| Aguirrezabal 2019 | Some concerns | Some concerns | High | Low | Some concerns | High |
| Alasfour 2022 | Low | Low | Some concerns | Some concerns | Low | Some concerns |
| Albaladejo 2010 | Low | Low | Low | Low | Low | Low |
| Almhdawi 2020 | High | Low | Some concerns | Low | Some concerns | High |
| Amaral 2020 | Low | Low | Low | Some concerns | Some concerns | Some concerns |
| Amer-Cuenca 2020 | Low | High | High | Low | Low | High |
| Archer 2016 | Low | Low | Low | Low | Low | Low |
| Areeudomwong 2017 | Low | Low | Low | Low | Some concerns | Some concerns |
| Ariza-Mateos 2020 | Some concerns | Low | Low | Low | Low | Some concerns |
| Bandak 2021 | Low | Low | Low | Low | Low | Low |
| Barrenengoa-Cuadra 2021 | Low | Low | Low | Some concerns | Low | Some concerns |
| Baumeister 2015 | Low | Low | Low | High | High | High |
| Beltran-Alacreu 2015 | Low | Low | Low | Low | Low | Low |
| Bennell 2017 | Some concerns | Low | Low | Some concerns | Low | Some concerns |
| Berube 2019 | Low | Low | Low | Some concerns | Low | Some concerns |
| BodesPardo 2018 | Low | Low | Low | Some concerns | Low | Some concerns |
| Bossen 2013 | Low | Low | Some concerns | Low | Low | Some concerns |
| Brison 2005 | Low | Some concerns | Low | Low | Some concerns | Some concerns |
| Burton 1999 | Low | Low | Low | Low | Low | Low |
| Chenot 2019 | Low | Some concerns | Some concerns | Some concerns | Low | Some concerns |
| Cherkin 2001 | Low | Low | Low | Low | Low | Low |
| Coudeyre 2006 | Low | Some concerns | High | Low | Some concerns | High |
| Coudeyre 2007 | Some concerns | Low | Low | Low | Low | Some concerns |
| Cramer 2013 | Some concerns | Low | Low | Low | Low | Some concerns |
| Cuesta-Vargas 2011 | Low | Low | Some concerns | Low | Low | Some concerns |
| Darnall 2020 | Low | Low | Low | Some concerns | Low | Some concerns |
| daSilva 2015 | Low | Low | Some concerns | Low | Low | Some concerns |
| Davis 2013 | Low | Low | Low | Some concerns | Low | Some concerns |
| deBoer 2014 | Low | Some concerns | Low | Some concerns | Some concerns | Some concerns |
| Derebery 2009 | Low | Some concerns | High | Some concerns | Some concerns | High |
| DiGiovanni 2003 | Some concerns | Some concerns | High | Some concerns | Low | High |
| Dobscha 2008 | Low | Some concerns | Some concerns | Low | Some concerns | Some concerns |
| Doering 2000 | Some concerns | Low | Some concerns | Low | Low | Some concerns |
| Dowd 2015 | Low | Some concerns | Low | Some concerns | Some concerns | Some concerns |
| Frost 1995 | Low | Low | Low | Low | Low | Low |
| Galan-Martin 2020 | Low | Low | Some concerns | Low | Low | Some concerns |
| Garcia 2021 | Low | Low | Low | Low | Low | Low |
| Garcia 2022 | Low | Low | Low | Low | Low | Low |
| Garcia-Palacios 2015 | Low | High | Some concerns | Some concerns | Low | Some concerns |
| Gardner 2019 | Low | Low | Low | Low | Low | Low |
| George 2009 | Some concerns | Low | Low | Some concerns | Low | Some concerns |
| Gibbs 2022 | Low | Low | Low | Some concerns | Some concerns | Some concerns |
| Giro 2016 | Low | Low | High | Low | Low | High |
| Grande-Alonso 2019 | Low | Low | Low | Low | Low | Some concerns |
| Hauser-Ulrich 2020 | Low | Low | Some concerns | Some concerns | Low | Some concerns |
| Heapy 2017 | Low | Low | Some concerns | Some concerns | Low | Some concerns |
| Hochlehnert 2006 | Low | Low | Low | Some concerns | Low | Some concerns |
| Ibrahim 2018 | Low | Low | Some concerns | Low | Low | Some concerns |
| Janevic 2022 | Some concerns | Low | Some concerns | High | Low | High |
| Jassi 2021 | Low | Some concerns | Some concerns | Low | Low | Some concerns |
| Javdaneh 2021 | Low | Low | Some concerns | Low | Some concerns | Some concerns |
| Jay 2014 | Low | Low | Low | Low | Low | Low |
| Khosrokiani 2022 | Low | Low | Low | Low | Low | Low |
| Kim 2021 | Some concerns | High | High | Low | Low | High |
| Kisaalita 2014 | Some concerns | Low | Low | Low | Some concerns | Some concerns |
| Ko 2013 |  |  |  |  |  |  |
| Kohns 2020 | Low | Some concerns | Low | Low | Some concerns | Some concerns |
| Kuvaƒçiƒá 2018 | Some concerns | Low | High | Some concerns | Low | High |
| Kwok 2016 | Some concerns | Low | Low | Some concerns | Some concerns | Some concerns |
| Lamb 2010 | Low | Low | Low | Low | Low | Low |
| Lefort 1998 | Low | Low | Low | Low | Low | Low |
| Li Y. 2020 | Low | Low | Low | Some concerns | Some concerns | Some concerns |
| Li Z. 2020 | Low | High | High | Some concerns | Some concerns | High |
| Lin 2018 | Low | High | Low | Some concerns | Low | High |
| Linton 2000 | Low | Some concerns | Some concerns | Some concerns | Some concerns | High |
| Little 2001 | Some concerns | Low | Some concerns | Some concerns | Some concerns | Some concerns |
| Lorig 2008 | High | Some concerns | Some concerns | Some concerns | Some concerns | High |
| Louw 2014 | Low | Low | Some concerns | Some concerns | Some concerns | Some concerns |
| Malfliet 2018 | Low | Low | Low | Low | Low | Some concerns |
| Marcus 2007 | Low | Some concerns | High | Some concerns | Some concerns | High |
| Mecklenburg 2018 | Some concerns | Low | Some concerns | Some concerns | Low | Some concerns |
| Meeus 2010 | Some concerns | Low | Low | Low | Some concerns | Some concerns |
| Mellor 2018 | Low | Low | Low | Low | Low | Low |
| Michaleff 2014 | Some concerns | Low | Low | Low | Low | Some concerns |
| Michou 2022 | Some concerns | Some concerns | Some concerns | Some concerns | Some concerns | High |
| Miyamoto 2018 | Low | Low | Low | Low | Low | Low |
| Morcillo-Mu√±oz 2022 | Low | Some concerns | High | Some concerns | High | High |
| Moseley 2002 | Low | Low | Some concerns | Low | Some concerns | Some concerns |
| Nambi 2020 | Some concerns | Low | Low | Some concerns | Some concerns | Some concerns |
| Nambi 2021 | Some concerns | Low | Low | Low | Some concerns | Some concerns |
| Nicholas 2017 | Low | Low | Some concerns | Low | Low | Some concerns |
| Nordin 2016 | Some concerns | Some concerns | Some concerns | Some concerns | Some concerns | High |
| O'Connor 2016 | High | Some concerns | High | Some concerns | High | High |
| O'Keeffe 2020 | Low | Some concerns | Low | Some concerns | Low | Some concerns |
| Oliveira 2006 | High | Some concerns | High | Some concerns | Some concerns | High |
| Pach 2022 | Some concerns | Some concerns | Some concerns | Some concerns | Low | Some concerns |
| Peters 2017 | High | Some concerns | Some concerns | Some concerns | Some concerns | High |
| Rabiei 2021 | Low | Some concerns | Some concerns | Some concerns | Some concerns | Some concerns |
| Rafiq 2021 | Some concerns | Low | Some concerns | Low | Low | Some concerns |
| Rini 2015 | Low | Low | Low | Some concerns | Some concerns | Some concerns |
| Riva 2014 | Some concerns | Low | Low | Low | Some concerns | Some concerns |
| Rodríguez-Torres 2020 | Low | Low | High | Some concerns | Some concerns | High |
| Ruehlman 2012 | Some concerns | Some concerns | Some concerns | Some concerns | Some concerns | High |
| Ryan 2010 | Some concerns | Some concerns | Some concerns | Some concerns | Some concerns | Some concerns |
| Rodríguez Sánchez-Laulhé 2022 | Low | Some concerns | Some concerns | Some concerns | Low | Some concerns |
| Saper 2017 | Some concerns | High | Some concerns | Low | Low | High |
| Saw 2016 | High | Some concerns | Some concerns | Some concerns | Some concerns | High |
| Schaller 2016 | High | Some concerns | Some concerns | Some concerns | Low | High |
| Schmidt 2021 | Low | Some concerns | Some concerns | Some concerns | Some concerns | High |
| Serrat 2021 | Low | Some concerns | High | Some concerns | Some concerns | High |
| Shaygan 2022 | Some concerns | High | High | Some concerns | High | High |
| Shaygan 2022 | Low | Some concerns | Low | Some concerns | Low | Some concerns |
| Sherman 2005 | Low | Low | Some concerns | Low | Some concerns | Some concerns |
| Sherman 2011 | Low | Low | Low | Some concerns | Low |  |
| Shpaner 2014 | Some concerns | Some concerns | Some concerns | Some concerns | Some concerns | High |
| Simister 2018 | Low | Low | Some concerns | Some concerns | Low | Some concerns |
| Simula 2021 | Some concerns | Some concerns | High | Some concerns | Some concerns | High |
| Singh 2018 | Some concerns | Low | Some concerns | Some concerns | Some concerns | High |
| Skillgate 2020 | Low | Low | Some concerns | Some concerns | Low | Some concerns |
| Skou 2015 | Low | Low | Low | Low | Low | Low |
| Skou 2015 | Low | Low | Low | Low | Low | Low |
| Sorensen 2010 | Low | Some concerns | Low | Low | Low | Some concerns |
| Syed 2018 | Some concerns | Low | Some concerns | Some concerns | Some concerns | Some concerns |
| Tejera 2020 | Low | Some concerns | Some concerns | Low | Some concerns | Some concerns |
| Thompson 2016 | Low | Low | Some concerns | Some concerns | Some concerns | Some concerns |
| Thorn 2018 | Low | Low | Low | Low | Low | Low |
| Timmerman 2016 | Some concerns | Some concerns | Low | Some concerns | Some concerns | Some concerns |
| Traeger 2019 | Low | Low | Low | Low | Low | Low |
| Triano 1995 | Low | Some concerns | Some concerns | Some concerns | Some concerns | Some concerns |
| Tse 2014 | High | High | High | Some concerns | Some concerns | High |
| Unal 2020 | Some concerns | Some concerns | Low | Some concerns | Some concerns | Some concerns |
| Valenza 2017 | Low | Some concerns | Low | Some concerns | Some concerns | Some concerns |
| Valiente-Castrillo 2021 | Some concerns | Some concerns | High | Some concerns | High | High |
| vanIttersum 2014 | Low | Low | Low | Low | Some concerns | Some concerns |
| VanOosterwijck 2013 | Low | Low | Low | Low | Some concerns | Some concerns |
| Vanti 2019 | Low | Some concerns | Low | Some concerns | High | High |
| Walsh 2020 | Low | Low | Low | Low | Low | Low |
| Wegwarth 2022 | Low | Low | Some concerns | Low | Low |  |
| Westenberg 2018 | Some concerns | Low | Low | Some concerns | High | High |
| Wiklund 2022 | Some concerns | Low | Low | Some concerns | Some concerns | Some concerns |
| Williams 2010 | Low | Low | Low | Some concerns | Some concerns | Some concerns |
| Yuan 2021 | Low | Low | Some concerns | Some concerns | Low | Some concerns |
| Zheng 2022 | High | Some concerns | Low | Low | Some concerns | High |
| Ziadni 2021 | Some concerns | Low | Low | Some concerns | Low | Some concerns |

**APPENDIX F - Reference list of included studies**

**N= [1-160]**

1. Aguirrezabal I, Pérez de San Román MS, Cobos-Campos R, Orruño E, Goicoechea A, Martínez de la Eranueva R, et al. Effectiveness of a primary care-based group educational intervention in the management of patients with migraine: a randomized controlled trial. Prim Health Care Res Dev. 2019 Dec 13;20:e155. PMID: 31833464. doi: 10.1017/s1463423619000720.

2. Alasfour M, Almarwani M. The effect of innovative smartphone application on adherence to a home-based exercise programs for female older adults with knee osteoarthritis in Saudi Arabia: a randomized controlled trial. Disabil Rehabil. 2022 Jun;44(11):2420-7. PMID: 33103499. doi: 10.1080/09638288.2020.1836268.

3. Albaladejo C, Kovacs FM, Royuela A, del Pino R, Zamora J. The efficacy of a short education program and a short physiotherapy program for treating low back pain in primary care: a cluster randomized trial. Spine (Phila Pa 1976). 2010 Mar 1;35(5):483-96. PMID: 20147875. doi: 10.1097/BRS.0b013e3181b9c9a7.

4. Almhdawi KA, Obeidat DS, Kanaan SF, Oteir AO, Mansour ZM, Alrabbaei H. Efficacy of an innovative smartphone application for office workers with chronic non-specific low back pain: a pilot randomized controlled trial. Clin Rehabil. 2020;34(10):1282-91. PMID: 32602362. doi: 10.1177/0269215520937757.

5. Amaral DDV, Miyamoto GC, Franco KFM, Dos Santos Franco YR, Bastos De Oliveira NT, Hancock MJ, et al. Examination of a Subgroup of Patients With Chronic Low Back Pain Likely to Benefit More From Pilates-Based Exercises Compared to an Educational Booklet. J Orthop Sports Phys Ther. 2020;50(4):189-97. PMID: 31443627. doi: 10.2519/jospt.2019.8839.

6. Amer-Cuenca JJ, Pecos-Martín D, Martínez-Merinero P, Lluch Girbés E, Nijs J, Meeus M, et al. How Much Is Needed? Comparison of the Effectiveness of Different Pain Education Dosages in Patients with Fibromyalgia. Pain Med. 2020 Apr 1;21(4):782-93. PMID: 31216027. doi: 10.1093/pm/pnz069.

7. Archer KR, Devin CJ, Vanston SW, Koyama T, Phillips SE, Mathis SL, et al. Cognitive-Behavioral-Based Physical Therapy for Patients With Chronic Pain Undergoing Lumbar Spine Surgery: A Randomized Controlled Trial. J Pain. 2016;17(1):76-89. PMID: 26476267. doi: 10.1016/j.jpain.2015.09.013.

8. Areeudomwong P, Wongrat W, Neammesri N, Thongsakul T. A randomized controlled trial on the long-term effects of proprioceptive neuromuscular facilitation training, on pain-related outcomes and back muscle activity, in patients with chronic low back pain. Musculoskeletal Care. 2017;15(3):218-29. PMID: 27791345. doi: 10.1002/msc.1165.

9. Ariza-Mateos MJ, Cabrera-Martos I, López-López L, Rodríguez-Torres J, Torres-Sánchez I, Valenza MC. Effects of a patient-centered program including the cumulative-complexity model in women with chronic pelvic pain: a randomized controlled trial. Maturitas. 2020;137:18-23. PMID: 32498932. doi: 10.1016/j.maturitas.2020.04.005.

10. Bandak E, Christensen R, Overgaard A, Kristensen LE, Ellegaard K, Guldberg-Møller J, et al. Exercise and education versus saline injections for knee osteoarthritis: a randomised controlled equivalence trial. Ann Rheum Dis. 2021. PMID: 34844929. doi: 10.1136/annrheumdis-2021-221129.

11. Barrenengoa-Cuadra MJ, Muñoa-Capron-Manieux M, Fernández-Luco M, Angón-Puras L, Romón-Gómez AJ, Azkuenaga M, et al. Effectiveness of a structured group intervention based on pain neuroscience education for patients with fibromyalgia in primary care: A multicentre randomized open-label controlled trial. Eur J Pain. 2021;25(5):1137-49. PMID: 33512028. doi: 10.1002/ejp.1738.

12. Baumeister H, Paganini S, Sander LB, Lin J, Schlicker S, Terhorst Y, et al. Effectiveness of a Guided Internet- and Mobile-Based Intervention for Patients with Chronic Back Pain and Depression (WARD-BP): A Multicenter, Pragmatic Randomized Controlled Trial. Psychother Psychosom. 2021;90(4):255-68. PMID: 33321501. doi: 10.1159/000511881.

13. Baumeister H, Seifferth H, Lin J, Nowoczin L, Lüking M, Ebert D. Impact of an Acceptance Facilitating Intervention on Patients' Acceptance of Internet-based Pain Interventions: A Randomized Controlled Trial. Clin J Pain. 2015;31(6):528-35. PMID: 24866854. doi: 10.1097/ajp.0000000000000118.

14. Beltran-Alacreu H, López-de-Uralde-Villanueva I, Fernández-Carnero J, La Touche R. Manual Therapy, Therapeutic Patient Education, and Therapeutic Exercise, an Effective Multimodal Treatment of Nonspecific Chronic Neck Pain: A Randomized Controlled Trial. Am J Phys Med Rehabil. 2015 Oct;94(10 Suppl 1):887-97. PMID: 25888653. doi: 10.1097/phm.0000000000000293.

15. Bennell KL, Nelligan R, Dobson F, Rini C, Keefe F, Kasza J, et al. Effectiveness of an Internet-Delivered Exercise and Pain-Coping Skills Training Intervention for Persons With Chronic Knee Pain: A Randomized Trial. Ann Intern Med. 2017;166(7):453-62. PMID: 28241215. doi: 10.7326/m16-1714.

16. Berberoğlu U, Ülger Ö. Multimedia Instructions for Motor Control Exercises in Patients With Chronic Nonspecific Low Back Pain. J Sport Rehabil. 2023 May 1;32(4):424-32. PMID: 36848900. doi: 10.1123/jsr.2022-0158.

17. Bérubé M, Gélinas C, Feeley N, Martorella G, Côté J, Laflamme GY, et al. Feasibility of a Hybrid Web-Based and In-Person Self-management Intervention Aimed at Preventing Acute to Chronic Pain Transition After Major Lower Extremity Trauma (iPACT-E-Trauma): A Pilot Randomized Controlled Trial. Pain Med. 2019;20(10):2018-32. PMID: 30840085. doi: 10.1093/pm/pnz008.

18. Bodes Pardo G, Lluch Girbés E, Roussel NA, Gallego Izquierdo T, Jiménez Penick V, Pecos Martín D. Pain Neurophysiology Education and Therapeutic Exercise for Patients With Chronic Low Back Pain: A Single-Blind Randomized Controlled Trial. Arch Phys Med Rehabil. 2018;99(2):338-47. PMID: 29138049. doi: 10.1016/j.apmr.2017.10.016.

19. Bossen D, Veenhof C, Van Beek KE, Spreeuwenberg PM, Dekker J, De Bakker DH. Effectiveness of a web-based physical activity intervention in patients with knee and/or hip osteoarthritis: randomized controlled trial. J Med Internet Res. 2013 Nov 22;15(11):e257. PMID: 24269911. doi: 10.2196/jmir.2662.

20. Bostrøm K, Børøsund E, Eide H, Varsi C, Kristjansdottir Ó B, Schreurs KMG, et al. Short-Term Findings From Testing EPIO, a Digital Self-Management Program for People Living With Chronic Pain: Randomized Controlled Trial. J Med Internet Res. 2023 Aug 25;25:e47284. PMID: 37624622. doi: 10.2196/47284.

21. Braun L, Terhorst Y, Titzler I, Freund J, Thielecke J, Ebert DD, et al. Lessons Learned from an Attempted Pragmatic Randomized Controlled Trial for Improvement of Chronic Pain-Associated Disability in Green Professions: Long-Term Effectiveness of a Guided Online-Based Acceptance and Commitment Therapy &nbsp;(PACT-A). Int J Environ Res Public Health. 2022 Oct 25;19(21). PMID: 36360738. doi: 10.3390/ijerph192113858.

22. Brison RJ, Hartling L, Dostaler S, Leger A, Rowe BH, Stiell I, et al. A randomized controlled trial of an educational intervention to prevent the chronic pain of whiplash associated disorders following rear-end motor vehicle collisions. Spine. 2005;30(16):1799-807. doi: 10.1097/01.brs.0000174115.58954.17.

23. Burton AK, Waddell G, Tillotson KM, Summerton N. Information and Advice to Patients With Back Pain Can Have a Positive Effect: A Randomized Controlled Trial of a Novel Educational Booklet in Primary Care. Spine. 1999;24(23):2484. PMID: 00007632-199912010-00010.

24. Chenot JF, Pfingsten M, Marnitz U, Pfeifer K, Kohlmann T, Lindena G, et al. [Effectiveness of a risk-tailored short intervention to prevent chronic low back pain : A cluster-randomized study in general practice]. Schmerz. 2019;33(3):226-35. PMID: 30796580. doi: 10.1007/s00482-019-0362-6.

25. Cherkin DC, Eisenberg D, Sherman KJ, Barlow W, Kaptchuk TJ, Street J, et al. Randomized trial comparing traditional Chinese medical acupuncture, therapeutic massage, and self-care education for chronic low back pain. Arch Intern Med. 2001 Apr 23;161(8):1081-8. PMID: 11322842. doi: 10.1001/archinte.161.8.1081.

26. Chimenti RL, Post AA, Rio EK, Moseley GL, Dao M, Mosby H, et al. The effects of pain science education plus exercise on pain and function in chronic Achilles tendinopathy: a blinded, placebo-controlled, explanatory, randomized trial. Pain. 2023 Jan 1;164(1):e47-e65. PMID: 36095045. doi: 10.1097/j.pain.0000000000002720.

27. Coudeyre E, Givron P, Vanbiervliet W, Benaim C, Herisson C, Pelissier J, et al. [The role of an information booklet or oral information about back pain in reducing disability and fear-avoidance beliefs among patients with subacute and chronic low back pain. A randomized controlled trial in a rehabilitation unit]. Ann Readapt Med Phys. 2006 Nov;49(8):600-8. PMID: 16793163. doi: 10.1016/j.annrmp.2006.05.003.

28. Coudeyre E, Tubach F, Rannou F, Baron G, Coriat F, Brin S, et al. Effect of a simple information booklet on pain persistence after an acute episode of low back pain: a non-randomized trial in a primary care setting. PLoS One. 2007;2(8):e706. PMID: 17684553. doi: 10.1371/journal.pone.0000706.

29. Cramer H, Lauche R, Hohmann C, Lüdtke R, Haller H, Michalsen A, et al. Randomized-controlled trial comparing yoga and home-based exercise for chronic neck pain. Clin J Pain. 2013;29(3):216-23. PMID: 23249655. doi: 10.1097/AJP.0b013e318251026c.

30. Cuesta-Vargas AI, García-Romero JC, Arroyo-Morales M, Diego-Acosta AM, Daly DJ. Exercise, manual therapy, and education with or without high-intensity deep-water running for nonspecific chronic low back pain: a pragmatic randomized controlled trial. Am J Phys Med Rehabil. 2011 Jul;90(7):526-34; quiz 35-8. PMID: 21765272. doi: 10.1097/PHM.0b013e31821a71d0.

31. da Silva FS, de Melo FES, do Amaral MMG, Caldas VVA, Pinheiro ILD, Abreu BJ, et al. Efficacy of simple integrated group rehabilitation program for patients with knee osteoarthritis: Single-blind randomized controlled trial. Journal of Rehabilitation Research and Development. 2015;52(3):309-21. PMID: WOS:000358111100006. doi: 10.1682/Jrrd.2014.08.0199.

32. Darnall BD, Krishnamurthy P, Tsuei J, Minor JD. Self-Administered Skills-Based Virtual Reality Intervention for Chronic Pain: Randomized Controlled Pilot Study. JMIR Form Res. 2020 Jul 7;4(7):e17293. PMID: 32374272. doi: 10.2196/17293.

33. Davis MC, Zautra AJ. An online mindfulness intervention targeting socioemotional regulation in fibromyalgia: results of a randomized controlled trial. Ann Behav Med. 2013 Dec;46(3):273-84. PMID: 23670111. doi: 10.1007/s12160-013-9513-7.

34. de Boer MJ, Versteegen GJ, Vermeulen KM, Sanderman R, Struys MM. A randomized controlled trial of an Internet-based cognitive-behavioural intervention for non-specific chronic pain: an effectiveness and cost-effectiveness study. Eur J Pain. 2014;18(10):1440-51. PMID: 24777973. doi: 10.1002/ejp.509.

35. Deegan O, Fullen BM, Casey MB, Segurado R, Hearty C, Doody C. Mindfulness Combined With Exercise Online (MOVE) Compared With a Self-management Guide for Adults With Chronic Pain: A Feasibility Randomized Controlled Trial. Clin J Pain. 2023 Aug 1;39(8):394-407. PMID: 37140219. doi: 10.1097/ajp.0000000000001126.

36. Derebery J, Giang GM, Gatchel RJ, Erickson K, Fogarty TW. Efficacy of a patient-educational booklet for neck-pain patients with workers' compensation: a randomized controlled trial. Spine (Phila Pa 1976). 2009 Jan 15;34(2):206-13. PMID: 19139673. doi: 10.1097/BRS.0b013e318193c9eb.

37. Diab R, Bomar R, Slaven J, Kaplan S, Ang D. Nurse-Supported Web-Based Cognitive Behavioral Therapy for Chronic Musculoskeletal Pain: A Randomized Controlled Trial. Pain Physician. 2022 Oct;25(7):E959-e68. PMID: 36288581.

38. DiGiovanni BF, Nawoczenski DA, Lintal ME, Moore EA, Murray JC, Wilding GE, et al. Tissue-specific plantar fascia-stretching exercise enhances outcomes in patients with chronic heel pain. A prospective, randomized study. J Bone Joint Surg Am. 2003 Jul;85(7):1270-7. PMID: 12851352. doi: 10.2106/00004623-200307000-00013.

39. Dobscha SK, Corson K, Leibowitz RQ, Sullivan MD, Gerrity MS. Rationale, design, and baseline findings from a randomized trial of collaborative care for chronic musculoskeletal pain in primary care. Pain Med. 2008;9(8):1050-64. PMID: 18565008. doi: 10.1111/j.1526-4637.2008.00457.x.

40. Doering S, Katzlberger F, Rumpold G, Roessler S, Hofstoetter B, Schatz DS, et al. Videotape preparation of patients before hip replacement surgery reduces stress. Psychosom Med. 2000 May-Jun;62(3):365-73. PMID: 10845350. doi: 10.1097/00006842-200005000-00010.

41. Dowd H, Hogan MJ, McGuire BE, Davis MC, Sarma KM, Fish RA, et al. Comparison of an Online Mindfulness-based Cognitive Therapy Intervention With Online Pain Management Psychoeducation: A Randomized Controlled Study. Clin J Pain. 2015 Jun;31(6):517-27. PMID: 25565584. doi: 10.1097/ajp.0000000000000201.

42. Fioratti I, Miyamoto GC, Fandim JV, Ribeiro CPP, Batista GD, Freitas GE, et al. Feasibility, Usability, and Implementation Context of an Internet-Based Pain Education and Exercise Program for Chronic Musculoskeletal Pain: Pilot Trial of the ReabilitaDOR Program. JMIR Form Res. 2022 Aug 30;6(8):e35743. PMID: 35776863. doi: 10.2196/35743.

43. Frost H, Klaber Moffett JA, Moser JS, Fairbank JCT. Randomised controlled trial for evaluation of fitness programme for patients with chronic low back pain. Br Med J. 1995;310(6973):151-4.

44. Galan-Martin MA, Montero-Cuadrado F, Lluch-Girbes E, Coca-López MC, Mayo-Iscar A, Cuesta-Vargas A. Pain Neuroscience Education and Physical Therapeutic Exercise for Patients with Chronic Spinal Pain in Spanish Physiotherapy Primary Care: A Pragmatic Randomized Controlled Trial. J Clin Med. 2020 Apr 22;9(4). PMID: 32331323. doi: 10.3390/jcm9041201.

45. Garcia LM, Birckhead BJ, Krishnamurthy P, Sackman J, Mackey IG, Louis RG, et al. An 8-week self-administered at-home behavioral skills-based virtual reality program for chronic low back pain: Double-blind, randomized, placebo-controlled trial conducted during COVID-19. J Med Internet Res. 2021;23(2). doi: 10.2196/26292.

46. Garcia-Palacios A, Herrero R, Vizcaíno Y, Belmonte MA, Castilla D, Molinari G, et al. Integrating virtual reality with activity management for the treatment of fibromyalgia: acceptability and preliminary efficacy. Clin J Pain. 2015 Jun;31(6):564-72. PMID: 25551475. doi: 10.1097/ajp.0000000000000196.

47. Gardner T, Refshauge K, McAuley J, Hübscher M, Goodall S, Smith L. Combined education and patient-led goal setting intervention reduced chronic low back pain disability and intensity at 12 months: a randomised controlled trial. Br J Sports Med. 2019;53(22):1424-31. PMID: 30808666. doi: 10.1136/bjsports-2018-100080.

48. Gasslander N, Andersson G, Boström F, Brandelius L, Pelling L, Hamrin L, et al. Tailored internet-based cognitive behavioral therapy for individuals with chronic pain and comorbid psychological distress: a randomized controlled trial. Cogn Behav Ther. 2022 Sep;51(5):408-34. PMID: 35533363. doi: 10.1080/16506073.2022.2065528.

49. George SZ, Teyhen DS, Wu SS, Wright AC, Dugan JL, Yang G, et al. Psychosocial education improves low back pain beliefs: results from a cluster randomized clinical trial (NCT00373009) in a primary prevention setting. Eur Spine J. 2009 Jul;18(7):1050-8. PMID: 19418075. doi: 10.1007/s00586-009-1016-7.

50. Gibbs MT, Morrison NM, Raftry S, Jones MD, Marshall PW. Does a powerlifting inspired exercise programme better compliment pain education compared to bodyweight exercise for people with chronic low back pain? A multicentre, single-blind, randomised controlled trial. Clin Rehabil. 2022 Sep;36(9):1199-213. PMID: 35466696. doi: 10.1177/02692155221095484.

51. Giro G, Policastro VB, Scavassin PM, Leite AR, Mendoza Marin DO, Gonçalves DA, et al. Mandibular kinesiographic pattern of women with chronic TMD after management with educational and self-care therapies: A double-blind, randomized clinical trial. J Prosthet Dent. 2016;116(5):749-55. PMID: 27236596. doi: 10.1016/j.prosdent.2016.03.021.

52. Grande-Alonso M, Suso-Martí L, Cuenca-Martínez F, Pardo-Montero J, Gil-Martínez A, La Touche R. Physiotherapy Based on a Biobehavioral Approach with or without Orthopedic Manual Physical Therapy in the Treatment of Nonspecific Chronic Low Back Pain: A Randomized Controlled Trial. Pain Medicine (United States). 2019;20(12):2571-87. doi: 10.1093/pm/pnz093.

53. Groenveld TD, Smits MLM, Knoop J, Kallewaard JW, Staal JB, de Vries M, et al. Effect of a Behavioral Therapy-Based Virtual Reality Application on Quality of Life in Chronic Low Back Pain. Clin J Pain. 2023 Jun 1;39(6):278-85. PMID: 37002877. doi: 10.1097/ajp.0000000000001110.

54. Hauser-Ulrich S, Künzli H, Meier-Peterhans D, Kowatsch T. A Smartphone-Based Health Care Chatbot to Promote Self-Management of Chronic Pain (SELMA): Pilot Randomized Controlled Trial. JMIR Mhealth Uhealth. 2020 Apr 3;8(4):e15806. PMID: 32242820. doi: 10.2196/15806.

55. Heapy AA, Higgins DM, Goulet JL, LaChappelle KM, Driscoll MA, Czlapinski RA, et al. Interactive Voice Response-Based Self-management for Chronic Back Pain: The COPES Noninferiority Randomized Trial. JAMA Intern Med. 2017;177(6):765-73. PMID: 28384682. doi: 10.1001/jamainternmed.2017.0223.

56. Hochlehnert A, Richter A, Bludau HB, Bieber C, Blumenstiel K, Mueller K, et al. A computer-based information-tool for chronic pain patients. Computerized information to support the process of shared decision-making. Patient Educ Couns. 2006;61(1):92-8. PMID: 16533681. doi: 10.1016/j.pec.2005.02.014.

57. Hrkać A, Bilić D, Černy-Obrdalj E, Baketarić I, Puljak L. Comparison of supervised exercise therapy with or without biopsychosocial approach for chronic nonspecific low back pain: a randomized controlled trial. BMC Musculoskelet Disord. 2022 Nov 8;23(1):966. PMID: 36348309. doi: 10.1186/s12891-022-05908-3.

58. Ibrahim AA, Akindele MO, Ganiyu SO. Motor control exercise and patient education program for low resource rural community dwelling adults with chronic low back pain: a pilot randomized clinical trial. J Exerc Rehabil. 2018 Oct;14(5):851-63. PMID: 30443533. doi: 10.12965/jer.1836348.174.

59. Ibrahim AA, Akindele MO, Ganiyu SO. Effectiveness of patient education plus motor control exercise versus patient education alone versus motor control exercise alone for rural community-dwelling adults with chronic low back pain: a randomised clinical trial. BMC Musculoskelet Disord. 2023 Feb 23;24(1):142. PMID: 36823567. doi: 10.1186/s12891-022-06108-9.

60. Janevic M, Robinson-Lane SG, Courser R, Brines E, Hassett AL. A Community Health Worker-Led Positive Psychology Intervention for African American Older Adults With Chronic Pain. Gerontologist. 2022 Oct 19;62(9):1369-80. PMID: 35394525. doi: 10.1093/geront/gnac010.

61. Jassi FJ, Del Antônio TT, Azevedo BO, Moraes R, George SZ, Chaves TC. Star-Shape Kinesio Taping Is Not Better Than a Minimal Intervention or Sham Kinesio Taping for Pain Intensity and Postural Control in Chronic Low Back Pain: A Randomized Controlled Trial. Arch Phys Med Rehabil. 2021;102(7):1352-60.e3. PMID: 33819489. doi: 10.1016/j.apmr.2021.03.007.

62. Javdaneh N, Saeterbakken AH, Shams A, Barati AH. Pain Neuroscience Education Combined with Therapeutic Exercises Provides Added Benefit in the Treatment of Chronic Neck Pain. Int J Environ Res Public Health. 2021 Aug 22;18(16). PMID: 34444594. doi: 10.3390/ijerph18168848.

63. Jay K, Schraefel MC, Brandt M, Andersen LL. Effect of video-based versus personalized instruction on errors during elastic tubing exercises for musculoskeletal pain: A randomized controlled trial. BioMed Research International. 2014;2014. doi: 10.1155/2014/790937.

64. Jinnouchi H, Kitamura A, Matsudaira K, Kakihana H, Oka H, Yamagishi K, et al. Brief self-exercise education for adults with chronic knee pain: A randomized controlled trial. Mod Rheumatol. 2023 Mar 2;33(2):408-15. PMID: 35134993. doi: 10.1093/mr/roac009.

65. Khosrokiani Z, Letafatkar A, Gladin A. Lumbar motor control training as a complementary treatment for chronic neck pain: A randomized controlled trial. Clin Rehabil. 2022 Jan;36(1):99-112. PMID: 34474578. doi: 10.1177/02692155211038099.

66. Kim SK, Kim HS, Chung SS. Effects of an Individualized Educational Program for Korean Patients With Chronic Low Back Pain: A Randomized Controlled Trial. J Nurs Res. 2021 Dec 1;29(6):e177. PMID: 34593721. doi: 10.1097/jnr.0000000000000455.

67. Kisaalita N, Staud R, Hurley R, Robinson M. Educational intervention about placebo mechanisms makes placebo use more acceptable for patients with chronic musculoskeletal pain. Journal of Pain. 2014;15(4):S118. doi: 10.1016/j.jpain.2014.01.481.

68. Ko V, Naylor J, Harris I, Crosbie J, Yeo A, Mittal R. One-to-one therapy is not superior to group or home-based therapy after total knee arthroplasty: a randomized, superiority trial. J Bone Joint Surg Am. 2013 Nov 6;95(21):1942-9. PMID: 24196464. doi: 10.2106/jbjs.L.00964.

69. Kohns DJ, Urbanik CP, Geisser ME, Schubiner H, Lumley MA. The Effects of a Pain Psychology and Neuroscience Self-Evaluation Internet Intervention: A Randomized Controlled Trial. Clin J Pain. 2020;36(9):683-92. PMID: 32520816. doi: 10.1097/ajp.0000000000000857.

70. Kuvačić G, Fratini P, Padulo J, Antonio DI, De Giorgio A. Effectiveness of yoga and educational intervention on disability, anxiety, depression, and pain in people with CLBP: A randomized controlled trial. Complement Ther Clin Pract. 2018;31:262-7. PMID: 29705466. doi: 10.1016/j.ctcp.2018.03.008.

71. Kwok EYT, Au RKC, Li-Tsang CWP. The effect of a self-management program on the quality-of-life of community-dwelling older adults with chronic musculoskeletal knee pain: A pilot randomized controlled trial. Clinical Gerontologist: The Journal of Aging and Mental Health. 2016;39(5):428-48. PMID: 1825958124; 2016-43562-006. doi: http://dx.doi.org/10.1080/07317115.2016.1171818.

72. Lamb SE, Hansen Z, Lall R, Castelnuovo E, Withers EJ, Nichols V, et al. Group cognitive behavioural treatment for low-back pain in primary care: a randomised controlled trial and cost-effectiveness analysis. Lancet. 2010;375(9718):916-23. doi: 10.1016/S0140-6736(09)62164-4.

73. Lefort SM, Gray-Donald K, Rowat KM, Jeans ME. Randomized controlled trial of a community-based psychoeducation program for the self-management of chronic pain. Pain. 1998;74(2-3):297-306. doi: 10.1016/S0304-3959(97)00190-5.

74. Li Y, Tse MYM. An Online Pain Education Program for Working Adults: Pilot Randomized Controlled Trial. J Med Internet Res. 2020 Jan 14;22(1):e15071. PMID: 31934865. doi: 10.2196/15071.

75. Li Z, Tse M, Tang A. The Effectiveness of a Dyadic Pain Management Program for Community-Dwelling Older Adults with Chronic Pain: A Pilot Randomized Controlled Trial. Int J Environ Res Public Health. 2020;17(14). PMID: 32660159. doi: 10.3390/ijerph17144966.

76. Lin J, Faust B, Ebert DD, Krämer L, Baumeister H. A Web-Based Acceptance-Facilitating Intervention for Identifying Patients' Acceptance, Uptake, and Adherence of Internet- and Mobile-Based Pain Interventions: Randomized Controlled Trial. J Med Internet Res. 2018;20(8):e244. PMID: 30131313. doi: 10.2196/jmir.9925.

77. Linton SJ, Andersson T, Linton SJ, Andersson T. Can chronic disability be prevented? A randomized trial of a cognitive-behavior intervention and two forms of information for patients with spinal pain. Spine (03622436). 2000;25(21):2825-31. PMID: 107005290. Language: English. Entry Date: 20010309. Revision Date: 20190818. Publication Type: journal article.

78. Little P, Roberts L, Blowers H, Garwood J, Cantrell T, Langridge J, et al. Should we give detailed advice and information booklets to patients with back pain? A randomized controlled factorial trial of a self-management booklet and doctor advice to take exercise for back pain. Spine (Phila Pa 1976). 2001 Oct 1;26(19):2065-72. PMID: 11698879. doi: 10.1097/00007632-200110010-00003.

79. Lorig KR, Ritter PL, Laurent DD, Plant K. The internet-based arthritis self-management program: a one-year randomized trial for patients with arthritis or fibromyalgia. Arthritis Rheum. 2008 Jul 15;59(7):1009-17. PMID: 18576310. doi: 10.1002/art.23817.

80. Louw A, Diener I, Landers MR, Puentedura EJ. Preoperative pain neuroscience education for lumbar radiculopathy: a multicenter randomized controlled trial with 1-year follow-up. Spine (Phila Pa 1976). 2014;39(18):1449-57. PMID: 24875964. doi: 10.1097/brs.0000000000000444.

81. Malfliet A, Kregel J, Coppieters I, De Pauw R, Meeus M, Roussel N, et al. Effect of Pain Neuroscience Education Combined With Cognition-Targeted Motor Control Training on Chronic Spinal Pain: A Randomized Clinical Trial. JAMA Neurol. 2018;75(7):808-17. PMID: 29710099. doi: 10.1001/jamaneurol.2018.0492.

82. Marcus BH, Lewis BA, Williams DM, Dunsiger S, Jakicic JM, Whiteley JA, et al. A comparison of Internet and print-based physical activity interventions. Arch Intern Med. 2007 May 14;167(9):944-9. PMID: 17502536. doi: 10.1001/archinte.167.9.944.

83. Mecklenburg G, Smittenaar P, Erhart-Hledik JC, Perez DA, Hunter S. Effects of a 12-Week Digital Care Program for Chronic Knee Pain on Pain, Mobility, and Surgery Risk: Randomized Controlled Trial. J Med Internet Res. 2018;20(4):e156. PMID: 29695370. doi: 10.2196/jmir.9667.

84. Meeus M, Nijs J, Van Oosterwijck J, Van Alsenoy V, Truijen S. Pain physiology education improves pain beliefs in patients with chronic fatigue syndrome compared with pacing and self-management education: A double-blind randomized controlled trial. Arch Phys Med Rehabil. 2010;91(8):1153-9. doi: 10.1016/j.apmr.2010.04.020.

85. Mellor R, Bennell K, Grimaldi A, Nicolson P, Kasza J, Hodges P, et al. Education plus exercise versus corticosteroid injection use versus a wait and see approach on global outcome and pain from gluteal tendinopathy: prospective, single blinded, randomised clinical trial. Bmj. 2018 May 2;361:k1662. PMID: 29720374. doi: 10.1136/bmj.k1662.

86. Michaleff ZA, Maher CG, Lin CW, Rebbeck T, Jull G, Latimer J, et al. Comprehensive physiotherapy exercise programme or advice for chronic whiplash (PROMISE): a pragmatic randomised controlled trial. Lancet. 2014;384(9938):133-41. PMID: 24703832. doi: 10.1016/s0140-6736(14)60457-8.

87. Michelotti A, Iodice G, Vollaro S, Steenks MH, Farella M. Evaluation of the short-term effectiveness of education versus an occlusal splint for the treatment of myofascial pain of the jaw muscles. J Am Dent Assoc. 2012 Jan;143(1):47-53. PMID: 22207667. doi: 10.14219/jada.archive.2012.0018.

88. Michou L, Julien AS, Witteman HO, Légaré J, Ratelle L, Godbout A, et al. Measuring the impact of an educational intervention in rheumatoid arthritis: An open-label, randomized trial. Arch Rheumatol. 2022 Jun;37(2):169-79. PMID: 36017212. doi: 10.46497/ArchRheumatol.2022.8965.

89. Miyamoto GC, Franco KFM, van Dongen JM, Franco Y, de Oliveira NTB, Amaral DDV, et al. Different doses of Pilates-based exercise therapy for chronic low back pain: a randomised controlled trial with economic evaluation. Br J Sports Med. 2018;52(13):859-68. PMID: 29525763. doi: 10.1136/bjsports-2017-098825.

90. Morcillo-Muñoz Y, Sánchez-Guarnido AJ, Calzón-Fernández S, Baena-Parejo I. Multimodal Chronic Pain Therapy for Adults via Smartphone: Randomized Controlled Clinical Trial. J Med Internet Res. 2022 May 11;24(5):e36114. PMID: 35373776. doi: 10.2196/36114.

91. Moseley L. Combined physiotherapy and education is efficacious for chronic low back pain. Aust J Physiother. 2002;48(4):297-302. PMID: 12443524. doi: 10.1016/s0004-9514(14)60169-0.

92. Mukhtar NB, Meeus M, Gursen C, Mohammed J, De Pauw R, Cagnie B. Pilot study on the effects of a culturally-sensitive and standard pain neuroscience education for Hausa-speaking patients with chronic neck pain. Disabil Rehabil. 2022 Nov;44(23):7226-36. PMID: 34663135. doi: 10.1080/09638288.2021.1988155.

93. Nambi G, Abdelbasset WK, Alrawaili SM, Alsubaie SF, Abodonya AM, Saleh AK. Virtual reality or isokinetic training; its effect on pain, kinesiophobia and serum stress hormones in chronic low back pain: A randomized controlled trial. Technol Health Care. 2021;29(1):155-66. PMID: 32831210. doi: 10.3233/thc-202301.

94. Nambi G, Abdelbasset WK, Elsayed SH, Alrawaili SM, Abodonya AM, Saleh AK, et al. Comparative Effects of Isokinetic Training and Virtual Reality Training on Sports Performances in University Football Players with Chronic Low Back Pain-Randomized Controlled Study. Evid Based Complement Alternat Med. 2020;2020:2981273. PMID: 32617104. doi: 10.1155/2020/2981273.

95. Nicholas MK, Asghari A, Blyth FM, Wood BM, Murray R, McCabe R, et al. Long-term outcomes from training in self-management of chronic pain in an elderly population: a randomized controlled trial. Pain. 2017;158(1):86-95. PMID: 27682207. doi: 10.1097/j.pain.0000000000000729.

96. Nordin CA, Michaelson P, Gard G, Eriksson MK. Effects of the Web Behavior Change Program for Activity and Multimodal Pain Rehabilitation: Randomized Controlled Trial. J Med Internet Res. 2016;18(10):e265. PMID: 27707686. doi: 10.2196/jmir.5634.

97. O'Connor MI, Brennan K, Kazmerchak S, Pratt J. YouTube Videos to Create a "Virtual Hospital Experience" for Hip and Knee Replacement Patients to Decrease Preoperative Anxiety: A Randomized Trial. Interact J Med Res. 2016;5(2):e10. PMID: 27091674. doi: 10.2196/ijmr.4295.

98. O'Keeffe M, O'Sullivan P, Purtill H, Bargary N, O'Sullivan K. Cognitive functional therapy compared with a group-based exercise and education intervention for chronic low back pain: a multicentre randomised controlled trial (RCT). Br J Sports Med. 2020;54(13):782-9. PMID: 31630089. doi: 10.1136/bjsports-2019-100780.

99. Oliveira A, Gevirtz R, Hubbard D. A psycho-educational video used in the emergency department provides effective treatment for whiplash injuries. Spine (Phila Pa 1976). 2006 Jul 1;31(15):1652-7. PMID: 16816758. doi: 10.1097/01.brs.0000224172.45828.e3.

100. Pacella-LaBarbara ML, Suffoletto BP, Kuhn E, Germain A, Jaramillo S, Repine M, et al. A Pilot Randomized Controlled Trial of the PTSD Coach App Following Motor Vehicle Crash-related Injury. Acad Emerg Med. 2020 Nov;27(11):1126-39. PMID: 32339359. doi: 10.1111/acem.14000.

101. Pach D, Blödt S, Wang J, Keller T, Bergmann B, Rogge AA, et al. App-Based Relaxation Exercises for Patients With Chronic Neck Pain: Pragmatic Randomized Trial. JMIR Mhealth Uhealth. 2022 Jan 7;10(1):e31482. PMID: 34994708. doi: 10.2196/31482.

102. Peters ML, Smeets E, Feijge M, van Breukelen G, Andersson G, Buhrman M, et al. Happy Despite Pain: A Randomized Controlled Trial of an 8-Week Internet-delivered Positive Psychology Intervention for Enhancing Well-being in Patients With Chronic Pain. Clin J Pain. 2017;33(11):962-75. PMID: 28379873. doi: 10.1097/ajp.0000000000000494.

103. Rabiei P, Sheikhi B, Letafatkar A. Comparing Pain Neuroscience Education Followed by Motor Control Exercises With Group-Based Exercises for Chronic Low Back Pain: A Randomized Controlled Trial. Pain Pract. 2021;21(3):333-42. PMID: 33135286. doi: 10.1111/papr.12963.

104. Rafiq MT, Hamid MSA, Hafiz E. Short-Term Effects of Strengthening Exercises of the Lower Limb Rehabilitation Protocol on Pain, Stiffness, Physical Function, and Body Mass Index among Knee Osteoarthritis Participants Who Were Overweight or Obese: A Clinical Trial. ScientificWorldJournal. 2021;2021:6672274. PMID: 34975349. doi: 10.1155/2021/6672274.

105. Rini C, Porter LS, Somers TJ, McKee DC, DeVellis RF, Smith M, et al. Automated Internet-based pain coping skills training to manage osteoarthritis pain: a randomized controlled trial. Pain. 2015 May;156(5):837-48. PMID: 25734997. doi: 10.1097/j.pain.0000000000000121.

106. Riva S, Camerini AL, Allam A, Schulz PJ. Interactive sections of an Internet-based intervention increase empowerment of chronic back pain patients: randomized controlled trial. J Med Internet Res. 2014;16(8):e180. PMID: 25119374. doi: 10.2196/jmir.3474.

107. Rodríguez Sánchez-Laulhé P, Luque-Romero LG, Barrero-García FJ, Biscarri-Carbonero Á, Blanquero J, Suero-Pineda A, et al. An Exercise and Educational and Self-management Program Delivered With a Smartphone App (CareHand) in Adults With Rheumatoid Arthritis of the Hands: Randomized Controlled Trial. JMIR Mhealth Uhealth. 2022 Apr 7;10(4):e35462. PMID: 35389367. doi: 10.2196/35462.

108. Rodríguez-Torres J, López-López L, Cabrera-Martos I, Prados-Román E, Granados-Santiago M, Valenza MC. Effects of an Individualized Comprehensive Rehabilitation Program on Impaired Postural Control in Women With Chronic Pelvic Pain: A Randomized Controlled Trial. Arch Phys Med Rehabil. 2020 Aug;101(8):1304-12. PMID: 32325162. doi: 10.1016/j.apmr.2020.02.019.

109. Roseen EJ, Pinheiro A, Lemaster CM, Plumb D, Wang S, Elwy AR, et al. Yoga Versus Education for Veterans with Chronic Low Back Pain: a Randomized Controlled Trial. J Gen Intern Med. 2023 Jul;38(9):2113-22. PMID: 36650329. doi: 10.1007/s11606-023-08037-2.

110. Ruehlman LS, Karoly P, Enders C. A randomized controlled evaluation of an online chronic pain self management program. Pain. 2012 Feb;153(2):319-30. PMID: 22133450. doi: 10.1016/j.pain.2011.10.025.

111. Ryan CG, Gray HG, Newton M, Granat MH. Pain biology education and exercise classes compared to pain biology education alone for individuals with chronic low back pain: A pilot randomised controlled trial. Man Ther. 2010;15(4):382-7. doi: 10.1016/j.math.2010.03.003.

112. Sandal LF, Bach K, Øverås CK, Svendsen MJ, Dalager T, Stejnicher Drongstrup Jensen J, et al. Effectiveness of App-Delivered, Tailored Self-management Support for Adults With Lower Back Pain-Related Disability: A selfBACK Randomized Clinical Trial. JAMA Intern Med. 2021 Oct 1;181(10):1288-96. PMID: 34338710. doi: 10.1001/jamainternmed.2021.4097.

113. Sander LB, Paganini S, Terhorst Y, Schlicker S, Lin J, Spanhel K, et al. Effectiveness of a Guided Web-Based Self-help Intervention to Prevent Depression in Patients With Persistent Back Pain: The PROD-BP Randomized Clinical Trial. JAMA Psychiatry. 2020 Oct 1;77(10):1001-11. PMID: 32459348. doi: 10.1001/jamapsychiatry.2020.1021.

114. Sandhu HK, Booth K, Furlan AD, Shaw J, Carnes D, Taylor SJC, et al. Reducing Opioid Use for Chronic Pain With a Group-Based Intervention: A Randomized Clinical Trial. Jama. 2023 May 23;329(20):1745-56. PMID: 37219554. doi: 10.1001/jama.2023.6454.

115. Saper RB, Lemaster C, Delitto A, Sherman KJ, Herman PM, Sadikova E, et al. Yoga, Physical Therapy, or Education for Chronic Low Back Pain: A Randomized Noninferiority Trial. Ann Intern Med. 2017;167(2):85-94. PMID: 28631003. doi: 10.7326/m16-2579.

116. Saw MM, Kruger-Jakins T, Edries N, Parker R. Significant improvements in pain after a six-week physiotherapist-led exercise and education intervention, in patients with osteoarthritis awaiting arthroplasty, in South Africa: A randomised controlled trial. BMC Musculoskeletal Disorders. 2016;17(1). doi: 10.1186/s12891-016-1088-6.

117. Schaller A, Dintsios CM, Icks A, Reibling N, Froboese I. Promoting physical activity in low back pain patients: six months follow-up of a randomised controlled trial comparing a multicomponent intervention with a low intensity intervention. Clin Rehabil. 2016;30(9):865-77. PMID: 27496696. doi: 10.1177/0269215515618730.

118. Schmidt S, Wölfle N, Schultz C, Sielmann D, Huber R, Walach H. Assessment of a taping method combined with manual therapy as a treatment of non-specific chronic low back pain - a randomized controlled trial. BMC Musculoskelet Disord. 2021;22(1):410. PMID: 33947367. doi: 10.1186/s12891-021-04236-2.

119. Serrat M, Albajes K, Navarrete J, Almirall M, Lluch Girbés E, Neblett R, et al. Effectiveness of two video-based multicomponent treatments for fibromyalgia: The added value of cognitive restructuring and mindfulness in a three-arm randomised controlled trial. Behav Res Ther. 2022 Nov;158:104188. PMID: 36116229. doi: 10.1016/j.brat.2022.104188.

120. Serrat M, Coll-Omaña M, Albajes K, Solé S, Almirall M, Luciano JV, et al. Efficacy of the FIBROWALK Multicomponent Program Moved to a Virtual Setting for Patients with Fibromyalgia during the COVID-19 Pandemic: A Proof-of-Concept RCT Performed Alongside the State of Alarm in Spain. Int J Environ Res Public Health. 2021;18(19). PMID: 34639600. doi: 10.3390/ijerph181910300.

121. Sharpe L, Jones EB, Pradhan P, Todd J, Colagiuri B. A double-blind phase II randomized controlled trial of an online cognitive bias modification for interpretation program with and without psychoeducation for people with chronic pain. Pain. 2023 Apr 1;164(4):e217-e27. PMID: 36607275. doi: 10.1097/j.pain.0000000000002784.

122. Shaygan M, Jaberi A, Firozian R, Yazdani Z. Comparing the effects of multimedia and face-to-face pain management education on pain intensity and pain catastrophizing among patients with chronic low back pain: A randomized clinical trial. PLoS One. 2022;17(6):e0269785. PMID: 35709207. doi: 10.1371/journal.pone.0269785.

123. Shaygan M, Jaberi A, Firozian R, Yazdani Z, Zarifsanaiey N. Effect of a multimedia training programme for pain management on pain intensity and depression in patients with non-specific chronic back pain. Invest Educ Enferm. 2022 Mar;40(1). PMID: 35485626. doi: 10.17533/udea.iee.v40n1e13.

124. Sherman KJ, Cherkin DC, Erro J, Miglioretti DL, Deyo RA. Comparing yoga, exercise, and a self-care book for chronic low back pain: a randomized, controlled trial. Ann Intern Med. 2005 Dec 20;143(12):849-56. PMID: 16365466. doi: 10.7326/0003-4819-143-12-200512200-00003.

125. Sherman KJ, Cherkin DC, Wellman RD, Cook AJ, Hawkes RJ, Delaney K, et al. A randomized trial comparing yoga, stretching, and a self-care book for chronic low back pain. Arch Intern Med. 2011 Dec 12;171(22):2019-26. PMID: 22025101. doi: 10.1001/archinternmed.2011.524.

126. Shpaner M, Kelly C, Lieberman G, Perelman H, Davis M, Keefe FJ, et al. Unlearning chronic pain: A randomized controlled trial to investigate changes in intrinsic brain connectivity following Cognitive Behavioral Therapy. Neuroimage Clin. 2014;5:365-76. PMID: 26958466. doi: 10.1016/j.nicl.2014.07.008.

127. Simister HD, Tkachuk GA, Shay BL, Vincent N, Pear JJ, Skrabek RQ. Randomized Controlled Trial of Online Acceptance and Commitment Therapy for Fibromyalgia. J Pain. 2018 Jul;19(7):741-53. PMID: 29481976. doi: 10.1016/j.jpain.2018.02.004.

128. Simula AS, Jenkins HJ, Hancock MJ, Malmivaara A, Booth N, Karppinen J. Patient education booklet to support evidence-based low back pain care in primary care - a cluster randomized controlled trial. BMC Fam Pract. 2021 Sep 7;22(1):178. PMID: 34493219. doi: 10.1186/s12875-021-01529-2.

129. Singh S, Clarke C, Lawendy AR, Macleod M, Sanders D, Tieszer C. First Place: A prospective, randomized controlled trial of the impact of written discharge instructions for postoperative opioids on patient pain satisfaction and on minimizing opioid risk exposure in orthopaedic surgery. Current Orthopaedic Practice. 2018;29(4):292-6. PMID: 130410036. Language: English. Entry Date: 20180703. Revision Date: 20210727. Publication Type: Article. doi: 10.1097/BCO.0000000000000632.

130. Skillgate E, Pico-Espinosa OJ, Côté P, Jensen I, Viklund P, Bottai M, et al. Effectiveness of deep tissue massage therapy, and supervised strengthening and stretching exercises for subacute or persistent disabling neck pain. The Stockholm Neck (STONE) randomized controlled trial. Musculoskelet Sci Pract. 2020;45:102070. PMID: 31655314. doi: 10.1016/j.msksp.2019.102070.

131. Skou ST, Rasmussen S, Laursen MB, Rathleff MS, Arendt-Nielsen L, Simonsen O, et al. The efficacy of 12 weeks non-surgical treatment for patients not eligible for total knee replacement: a randomized controlled trial with 1-year follow-up. Osteoarthritis Cartilage. 2015;23(9):1465-75. PMID: 25937024. doi: 10.1016/j.joca.2015.04.021.

132. Skou ST, Roos EM, Laursen MB, Rathleff MS, Arendt-Nielsen L, Simonsen O, et al. A Randomized, Controlled Trial of Total Knee Replacement. N Engl J Med. 2015;373(17):1597-606. PMID: 26488691. doi: 10.1056/NEJMoa1505467.

133. Sorensen PH, Bendix T, Manniche C, Korsholm L, Lemvigh D, Indahl A. An educational approach based on a non-injury model compared with individual symptom-based physical training in chronic LBP. A pragmatic, randomised trial with a one-year follow-up. BMC Musculoskeletal Disorders. 2010;11. doi: 10.1186/1471-2474-11-212.

134. Syed UAM, Aleem AW, Wowkanech C, Weekes D, Freedman M, Tjoumakaris F, et al. Neer Award 2018: the effect of preoperative education on opioid consumption in patients undergoing arthroscopic rotator cuff repair: a prospective, randomized clinical trial. J Shoulder Elbow Surg. 2018;27(6):962-7. PMID: 29599038. doi: 10.1016/j.jse.2018.02.039.

135. Tejera DM, Beltran-Alacreu H, Cano-de-la-Cuerda R, Leon Hernández JV, Martín-Pintado-Zugasti A, Calvo-Lobo C, et al. Effects of Virtual Reality versus Exercise on Pain, Functional, Somatosensory and Psychosocial Outcomes in Patients with Non-specific Chronic Neck Pain: A Randomized Clinical Trial. Int J Environ Res Public Health. 2020 Aug 16;17(16). PMID: 32824394. doi: 10.3390/ijerph17165950.

136. Thompson DP, Oldham JA, Woby SR. Does adding cognitive-behavioural physiotherapy to exercise improve outcome in patients with chronic neck pain? A randomised controlled trial. Physiotherapy. 2016 Jun;102(2):170-7. PMID: 26383695. doi: 10.1016/j.physio.2015.04.008.

137. Thorn BE, Eyer JC, Van Dyke BP, Torres CA, Burns JW, Kim M, et al. Literacy-Adapted Cognitive Behavioral Therapy Versus Education for Chronic Pain at Low-Income Clinics: A Randomized Controlled Trial. Ann Intern Med. 2018;168(7):471-80. PMID: 29482213. doi: 10.7326/m17-0972.

138. Timmerman L, Stronks DL, Groeneweg G, Huygen FJ. The Value of Medication-Specific Education on Medication Adherence and Treatment Outcome in Patients with Chronic Pain: A Randomized Clinical Trial. Pain Med. 2016;17(10):1829-37. PMID: 26921890. doi: 10.1093/pm/pnw013.

139. Traeger AC, Lee H, Hübscher M, Skinner IW, Moseley GL, Nicholas MK, et al. Effect of Intensive Patient Education vs Placebo Patient Education on Outcomes in Patients with Acute Low Back Pain: A Randomized Clinical Trial. JAMA Neurology. 2019;76(2):161-9. doi: 10.1001/jamaneurol.2018.3376.

140. Triano JJ, McGregor M, Hondras MA, Brennan PC, Triano JJ, McGregor M, et al. Manipulative therapy versus education programs in chronic low back pain. Spine (03622436). 1995;20(8):948-55. PMID: 107428349. Language: English. Entry Date: 19951101. Revision Date: 20190818. Publication Type: journal article.

141. Tse MM, Tang SK, Wan VT, Vong SK. The effectiveness of physical exercise training in pain, mobility, and psychological well-being of older persons living in nursing homes. Pain Manag Nurs. 2014;15(4):778-88. PMID: 24361207. doi: 10.1016/j.pmn.2013.08.003.

142. Tse MMY, Yan E, Tang ASK, Cheung D, Ng S. A music-with-movement exercise programme for community-dwelling older adults suffering from chronic pain: A pilot randomized controlled trial. Nurs Open. 2023 Sep;10(9):6566-74. PMID: 37415289. doi: 10.1002/nop2.1915.

143. Ünal M, Evci KE, Kocatürk M, Algun ZC. Investigating the effects of myofascial induction therapy techniques on pain, function and quality of life in patients with chronic low back pain. J Bodyw Mov Ther. 2020;24(4):188-95. PMID: 33218510. doi: 10.1016/j.jbmt.2020.07.014.

144. Valenza MC, Rodríguez-Torres J, Cabrera-Martos I, Díaz-Pelegrina A, Aguilar-Ferrándiz ME, Castellote-Caballero Y. Results of a Pilates exercise program in patients with chronic non-specific low back pain: a randomized controlled trial. Clin Rehabil. 2017;31(6):753-60. PMID: 27260764. doi: 10.1177/0269215516651978.

145. Valiente-Castrillo P, Martín-Pintado-Zugasti A, Calvo-Lobo C, Beltran-Alacreu H, Fernández-Carnero J. Effects of pain neuroscience education and dry needling for the management of patients with chronic myofascial neck pain: a randomized clinical trial. Acupunct Med. 2021;39(2):91-105. PMID: 32370545. doi: 10.1177/0964528420920300.

146. van Ittersum MW, van Wilgen CP, van der Schans CP, Lambrecht L, Groothoff JW, Nijs J. Written Pain Neuroscience Education in Fibromyalgia: A Multicenter Randomized Controlled Trial. Pain Practice. 2014;14(8):689-700. doi: 10.1111/papr.12137.

147. Van Oosterwijck J, Meeus M, Paul L, De Schryver M, Pascal A, Lambrecht L, et al. Pain physiology education improves health status and endogenous pain inhibition in fibromyalgia: a double-blind randomized controlled trial. Clin J Pain. 2013;29(10):873-82. PMID: 23370076. doi: 10.1097/AJP.0b013e31827c7a7d.

148. Vanti C, Banchelli F, Marino C, Puccetti A, Guccione AA, Pillastrini P. Effectiveness of a "Spring Pillow" Versus Education in Chronic Nonspecific Neck Pain: A Randomized Controlled Trial. Phys Ther. 2019;99(9):1177-88. PMID: 30939188. doi: 10.1093/ptj/pzz056.

149. Vicente-Mampel J, Gargallo P, Bautista IJ, Blanco-Gímenez P, de Bernardo Tejedor N, Alonso-Martín M, et al. Impact of Pain Neuroscience Education Program in Community Physiotherapy Context on Pain Perception and Psychosocial Variables Associated with It in Elderly Persons: A Ranzomized Controlled Trial. Int J Environ Res Public Health. 2022 Sep 20;19(19). PMID: 36231171. doi: 10.3390/ijerph191911855.

150. Walsh N, Jones L, Phillips S, Thomas R, Odondi L, Palmer S, et al. Facilitating Activity and Self-management for people with Arthritic knee, hip or lower back pain (FASA): A cluster randomised controlled trial. Musculoskelet Sci Pract. 2020;50. doi: 10.1016/j.msksp.2020.102271.

151. Wegwarth O, Ludwig WD, Spies C, Schulte E, Hertwig R. The role of simulated-experience and descriptive formats on perceiving risks of strong opioids: A randomized controlled trial with chronic noncancer pain patients. Patient Educ Couns. 2022 Jun;105(6):1571-80. PMID: 34696941. doi: 10.1016/j.pec.2021.10.002.

152. Westenberg RF, Zale EL, Heinhuis TJ, Ozkan S, Nazzal A, Lee SG, et al. Does a brief mindfulness exercise improve outcomes in upper extremity patients? A randomized controlled trial. Clinical Orthopaedics and Related Research. 2018;476(4):790-8. doi: 10.1007/s11999.0000000000000086.

153. Wiklund T, Molander P, Lindner P, Andersson G, Gerdle B, Dragioti E. Internet-Delivered Cognitive Behavioral Therapy for Insomnia Comorbid With Chronic Pain: Randomized Controlled Trial. J Med Internet Res. 2022 Apr 29;24(4):e29258. PMID: 35486418. doi: 10.2196/29258.

154. Williams DA, Kuper D, Segar M, Mohan N, Sheth M, Clauw DJ. Internet-enhanced management of fibromyalgia: a randomized controlled trial. Pain. 2010 Dec;151(3):694-702. PMID: 20855168. doi: 10.1016/j.pain.2010.08.034.

155. Williams RM, Day MA, Ehde DM, Turner AP, Ciol MA, Gertz KJ, et al. Effects of hypnosis vs mindfulness meditation vs education on chronic pain intensity and secondary outcomes in veterans: a randomized clinical trial. Pain. 2022 Oct 1;163(10):1905-18. PMID: 35082248. doi: 10.1097/j.pain.0000000000002586.

156. Wilson M, Dolor RJ, Lewis D, Regan SL, Vonder Meulen MB, Winhusen TJ. Opioid dose and pain effects of an online pain self-management program to augment usual care in adults with chronic pain: a multisite randomized clinical trial. Pain. 2023 Apr 1;164(4):877-85. PMID: 36525381. doi: 10.1097/j.pain.0000000000002785.

157. Yeh CH, Kawi J, Grant L, Huang X, Wu H, Hardwicke RL, et al. Self-Guided Smartphone Application to Manage Chronic Musculoskeletal Pain: A Randomized, Controlled Pilot Trial. Int J Environ Res Public Health. 2022 Nov 11;19(22). PMID: 36429591. doi: 10.3390/ijerph192214875.

158. Yuan SLK, Couto LA, Marques AP. Effects of a six-week mobile app versus paper book intervention on quality of life, symptoms, and self-care in patients with fibromyalgia: a randomized parallel trial. Braz J Phys Ther. 2021;25(4):428-36. PMID: 33248904. doi: 10.1016/j.bjpt.2020.10.003.

159. Zheng F, Liu S, Zhang S, Yu Q, Lo WLA, Li T, et al. Does m-health-based exercise (guidance plus education) improve efficacy in patients with chronic low-back pain? A preliminary report on the intervention's significance. Trials. 2022 Mar 3;23(1):190. PMID: 35241140. doi: 10.1186/s13063-022-06116-z.

160. Ziadni MS, Gonzalez-Castro L, Anderson S, Krishnamurthy P, Darnall BD. Efficacy of a Single-Session "Empowered Relief" Zoom-Delivered Group Intervention for Chronic Pain: Randomized Controlled Trial Conducted During the COVID-19 Pandemic. J Med Internet Res. 2021;23(9):e29672. PMID: 34505832. doi: 10.2196/29672.
